# Molecular docking, free energy calculations, ADMETox studies, DFT analysis, and dynamic simulations highlighting a chromene glycoside as a potential inhibitor of PknG in *Mycobacterium tuberculosis*

**DOI:** 10.3389/fchem.2025.1531152

**Published:** 2025-02-25

**Authors:** Muharib Alruwaili, Tilal Elsaman, Magdi Awadalla Mohamed, Abozer Y. Elderdery, Jeremy Mills, Yasir Alruwaili, Siddiqa M. A. Hamza, Salma Elhadi Ibrahim Mekki, Hazim Abdullah Alotaibi, Maily J. Alrowily, Maryam Musleh Althobiti

**Affiliations:** ^1^ Department of Clinical Laboratory Sciences, College of Applied Medical Sciences, Jouf University, Sakaka, Saudi Arabia; ^2^ Department of Pharmaceutical Chemistry, College of Pharmacy, Jouf University, Sakaka, Saudi Arabia; ^3^ School of Pharmacy and Biomedical Sciences, University of Portsmouth, Portsmouth, United Kingdom; ^4^ Department of Pathology, College of Medicine in Alqunfudah, Umm Alqura University, Algunfuda, Saudi Arabia; ^5^ Department of Physiology, College of Medicine in Alqunfudah, Umm Alqura University, Alqunfudah, Saudi Arabia; ^6^ Department of Internal Medicine and Oncology, Prince Mohammed Medical City, Hail, Saudi Arabia; ^7^ Consultant -Research Center, Aljouf Health Cluster, Aljouf, Saudi Arabia; ^8^ Department of Clinical Laboratory Sciences, College of Applied Medical Sciences, Shaqra University, Shaqra, Saudi Arabia

**Keywords:** *Mycobacterium tuberculosis*, PknG, resistance, multidrug resistant-TB (MDR-TB), extensively drug resistant-TB (XDR-TB)

## Abstract

**Introduction:**

Tuberculosis (TB), caused by the *Mycobacterium tuberculosis* (M.tb), remains a serious medical concern globally. Resistant M.tb strains are emerging, partly because M.tb can survive within alveolar macrophages, resulting in persistent infection. Protein kinase G (PknG) is a mycobacterial virulence factor that promotes the survival of M.tb in macrophages. Targeting PknG could offer an opportunity to suppress the resistant M.tb strains.

**Methods:**

In the present study, multiple computational tools were adopted to screen a library of 460,000 molecules for potential inhibitors of PknG of M.tb.

**Results and discussions:**

Seven Hits (**1–7**) were identified with binding affinities exceeding that of the reference compound (AX20017) towards the PknG catalytic domain. Next, the ADMETox studies were performed to identify the best hit with appropriate drug-like properties. The chromene glycoside (Hit **1**) was identified as a potential PknG inhibitor with better pharmacokinetic and toxicity profiles rendering it a potential drug candidate. Furthermore, quantum computational analysis was conducted to assess the mechanical and electronic properties of Hit **1,** providing guidance for further studies. Molecular dynamics simulations were also performed for Hit **1** against PknG, confirming the stability of its complex. In sum, the findings in the current study highlight Hit **1** as a lead with potential for development of drugs capable of treating resistant TB.

## 1 Introduction

**FIGURE 1:**
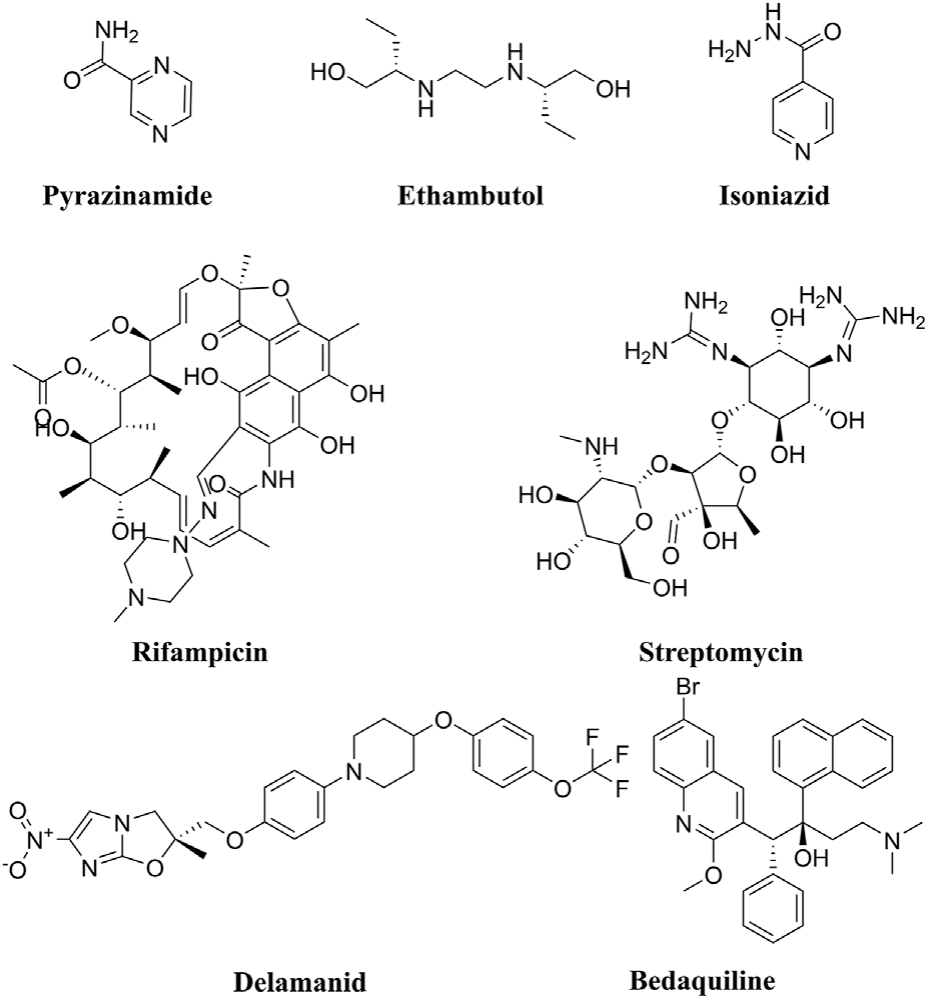
Chemical structures of the front-line anti-TB drugs (Pyrazinamide, Ethambutol, Isoniazid, Rifampicin, and Streptomycin). In addition, the chemical structures of the recently approved anti-TB drugs Delamanid and Bedaquiline are also depicted.

Tuberculosis (TB) is a contagious illness caused by the airborne bacilli *Mycobacterium tuberculosis* (M.tb). Its primary site of infection is the lung, but it can involve other organs in the human body. Some studies reported that TB is still a serious global health concern, particularly in the global south (Fernandes et al., 2022; Bloom, 2023). In 2020, the estimated number of symptomatic new TB cases was 9.9 million, with over 1.5 million deaths (Bagcchi, 2023). Fernandes, G.F.S and co-workers reported high morbidity and mortality rates in TB patients around the globe (Fernandes et al., 2022). In fact, from 2012 to 2019, TB was the leading cause of death from a single infectious agent (Friedrich, 2017; WHO, 2020). Since 1960, successful efforts have been made to develop a variety of antibiotics (Figure 1) which have contributed to the saving of millions of human lives. However, the number of currently used drugs in clinical practice is very limited and this has been further exacerbated by the emergence of resistant strains of TB, such as multidrug resistant TB (MDR-TB) and extensive drug resistant TB (XDR-TB) (Dartois and Rubin, 2022). Moreover, resistant strains to newly approved anti-TB medications, including Delamanid and Bedaquiline (Figure 1) have been detected in clinical settings (Yoshiyama et al., 2021).

**FIGURE 2:**
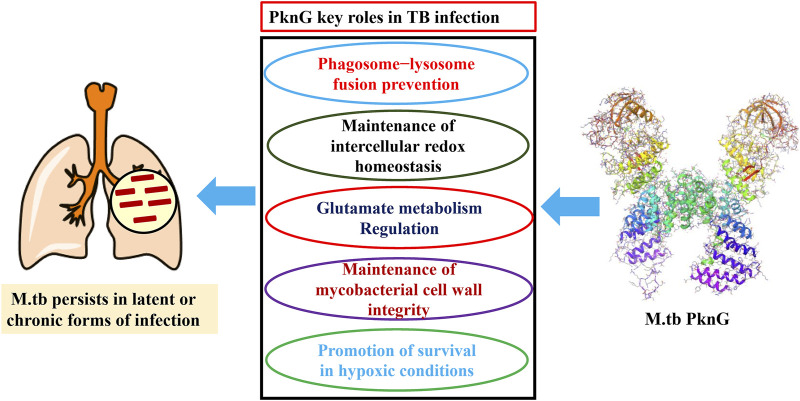
Mechanisms by which PknG enhances the *in vivo* survival and persistence of M.tb.

This troubling condition is due, to some extent, to a nearly 5 decade long gap in anti-TB medication discovery research and development (Teneva et al., 2023). In general, the current TB chemotherapy is often inadequate due to multiple factors including (i) the prolonged treatment regiments; (ii) serious adverse effects, (iii) the continued evolution of drug resistance, and (iv) the slow development of new therapeutics (Rock, 2019; Akinnuwesi et al., 2023; Capela et al., 2023). To this end, mycobacterial resistance to the currently used anti-TB drugs has been attributed, in part, to the ability of the M.tb to survive within macrophages resulting in persistent infection (latent TB) (Simmons et al., 2018). Multiple research studies have demonstrated that the virulence factor protein kinase G (PknG), which contains a thioredoxin motif, aids in the survival of M.tb inside macrophages through several different mechanisms (Figure 2) (Koul et al., 2001; Cowley et al., 2004; O'Hare et al., 2008; Forrellad et al., 2013; Khan et al., 2017; Swain et al., 2022). Therefore, PknG could be considered as a crucial druggable macromolecule for the development of new therapeutics with potential to both, inhibit non-proliferating mycobacteria and suppress the evolution of M.tb resistant strains (Khan et al., 2018). Recently, several molecules belonging to secondary plant metabolites have been investigated as potential anti-tubercular agents, including diarylheptanoids, 3-glycosyl isocoumarins, biphenyl and diaryl ether diarylheptanoids, tetrahydropyran-based diarylheptanoids such as engelheptanoxides, and various 3-aryl isocoumarins (Sudarshan and Aidhen, 2017; Sudarshan et al., 2024).

Computational methods accelerate drug discovery by identifying and optimizing therapeutic agents while reducing time, cost, and experimental efforts. They provide key insights into molecular interactions and target validation (Athar et al., 2016). Exploring the structural targets of M.tb through *in silico* approaches aids in identifying promising therapeutic candidates. This method enhances the understanding of protein structures and interactions, streamlining the process of tuberculosis drug discovery (Lone et al., 2018).

**FIGURE 3:**
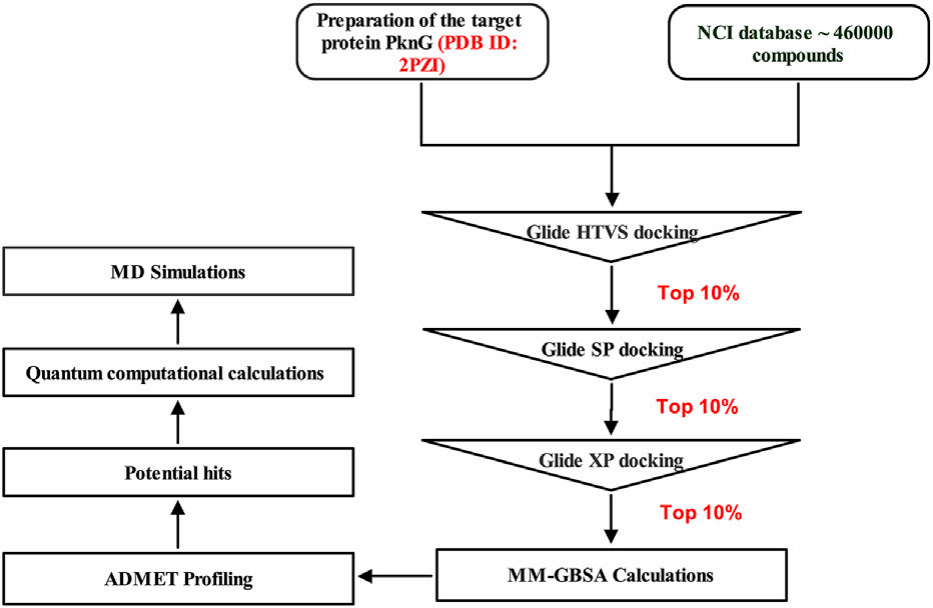
Multistep virtual screening workflow for the identification of potential PknG inhibitors.

In this study, we screened the NCI library (https://cactus.nci.nih.gov/download/roadmap/) of 460,000 molecules using the CADD approach (Figure 3) [20] against the PknG ATP binding pocket, aiming to identify novel inhibitors as potential leads against TB.

## 2 Materials and methods

In this work various computational tools interfaced in Schrödinger suite were used, including Protein Preparation Wizard (Sastry et al., 2013), LigPrep (Schrödinger Release, 2023), Glide (Friesner et al., 2004; Halgren et al., 2024), Prime (Jacobson et al., 2002a; Jacobson et al., 2002b), Qikprop (QikProp, 2023), Jaguar (Bochevarov et al., 2013) and Desmond (Bowers et al., 2006). These tools were accessed utilizing Maestro graphical interface (Maestro, 2023). Additionally, ADMETlab 2.0 (Xiong et al., 2021) an integrated online webserver was used for ADMET properties estimation.

### 2.1 Molecular docking study

Virtual screening analysis of the NCI library (https://cactus.nci.nih.gov/download/roadmap/) of 460,000 molecules was conducted, using Virtual Screening Workflow (VSW) of Schrödinger suite. The downloaded compounds library were converted to 3D formats and then OPLS4 force field was used for optimization. The structures were set for docking and the ligands’ original chirality’s were maintained, using LigPrep module. For generating the possible ionization states, the Epik was used at pH 7.00 ± 2 units. For each ligand one low energy conformer was generated. The 3D crystal structure of M.tb PknG complexed with the reference ligand AX20017 (PDB code: 2PZI), was obtained from the Protein Data Bank (PDB) website (www.rcsb.org). Subsequently, we utilized the multi-step Protein Preparation Wizard (PrepWizard) to refine and optimize the protein structure for further analysis. After removal of all water molecules, only the co-crystallized ligand remained at the enzyme’s catalytic site. OPLS4 force field was used to perform optimization and energy minimization. The receptor grid generation tool implanted within the maestro suite was employed to create the grid box around the coordinates of the reference ligand. Prepared ligands were then screened against the refined target protein following multimode receptor docking workflow using Glide module of Schrödinger suite. Initially, Glide high-throughput virtual screening (HTVS) mode was employed for filtering the compounds library, then for further screening the standard precision (SP) mode was used and finally more accurate docking calculations results were obtained utilizing extra precision (XP) mode. A single optimal pose was generated for each input molecule, and their ranking was decided based on their Glide docking score.

### 2.2 Binding free energy calculations

Estimation of the binding free energies of receptors and docked ligands was done using Prime module. The free binding energy of the protein-ligand complexes was computed using the Prime module, integrated with Schrödinger software. For this purpose, the Post-docking generated Pose Viewer Files (PVFs) of the top Hits**1-7** were used as input files. Free binding energy parameters were then calculated using OPLS4 (Optimized Potentials for Liquid Simulations, version 4) force field along with Variable Dielectric Generalized Born (VSGB) 2.0 as solvation model, following the established protocol (Azam et al., 2021). Molecular Mechanics-Generalized Born Surface Area (MM-GBSA) dG binding energy score was employed to rank the ligands based on their relative affinities.

### 2.3 *In silico* absorption, distribution, metabolism, excretion, and toxicity (ADMET) profiling

In this study, we evaluated the ADMET profiles and drug-likeness characteristics of the top 7 candidates using the Qikprop computational tool within the Schrödinger suite. Furthermore, the free web-based tool ADMETlab 2.0 available at (https://admetmesh.scbdd.com) was employed to predict the toxicity parameters. All of the calculated parameters were evaluated to ensure compliance with their respective standard ranges.

### 2.4 Quantum computational calculations

Quantum chemical calculations of the co-crystal ligand and Hit **1** electronic molecular properties such as Electron density, Molecular Electrostatic Potential Map (MESP) and energies of both Highest Occupied Molecular Orbital (HOMO) and Lowest Unoccupied Molecular Orbital (LUMO) were calculated using DFT method in the Jaguar module of Schrödinger suite (Khan and Singh, 2023). The values of the E_LUMO_ and E_HOMO_ were subsequently employed to compute the various quantum chemical properties, including the energy gap HOMO-LUMO Gab (HLG), chemical chemicals (softness and hardness), global electrophilicity index and electronegativity, according to equations established in the literature (Kohn and Sham, 1965; Guezane-Lakoud et al., 2023). Electron-deficient surfaces are marked by the blue color, whereas the electron-rich ones are indicated by the red color.

### 2.5 Molecular dynamics (MD) simulations

MD simulations were achieved using Desmond. The input files of docked conformers of both Hit **1** and the co-crystallized ligand complexes were obtained from docking study. These complexes were immersed in a cubic water box with dimensions of 10 Å × 10 Å × 10 Å, utilizing Simple Point Charge (SPC) as solvation medium. To achieve balance in the net charges, Na^+^ counter ions were added to the built systems, and subsequently sodium chloride 0.15 M was also added to attain the system’s neutralization. The simulations were conducted in an NPT ensemble, ensuring that the temperature and pressure of the system were maintained at 300 K and 1 bar, respectively. Prior to simulation run, the default relaxation protocol in the Desmond module of Schrodinger suite 2023-1 was then used to minimize and pre-equilibrate the system. The MD simulations extended over a duration of 100 ns, generating 1,000 frames of data captured at every 100 ps interval. The Simulation Interaction Diagram tool in Desmond was used to perform post-simulation trajectory analysis. Critical parameters such as Root Mean Square Deviation (RMSD), Root Mean Square Fluctuation (RMSF), and protein-ligand contacts were computed to assess the stability of the complexes and the nature of their interaction profiles throughout the simulation period.

## 3 Results and discussion

Despite the existence of several drugs for treatment of TB infection, drawbacks associated with them such as prolonged treatment regimens, adverse effects, poor patient compliance and emergence of drug resistance, dictates the development of novel drugs with optimum therapeutic properties (Rock, 2019; Akinnuwesi et al., 2023; Capela et al., 2023). An *in silico* strategy is considered an attractive approach that could be applied at different drug discovery stages to accelerate the identification of potential anti-TB drug leads (Macalino et al., 2020). In the current work, we report on high-throughput multilevel virtual screening of 460,000 molecules from an NCI database against the ATP binding site of PknG to identify potential hits using molecular docking score and the free binding energy to as filtering parameters. Further, ADMET profiling and quantum computational calculations were performed to better understand their drug utility and to discriminate between hits for further optimization studies.

### 3.1 Molecular docking study

**FIGURE 4:**
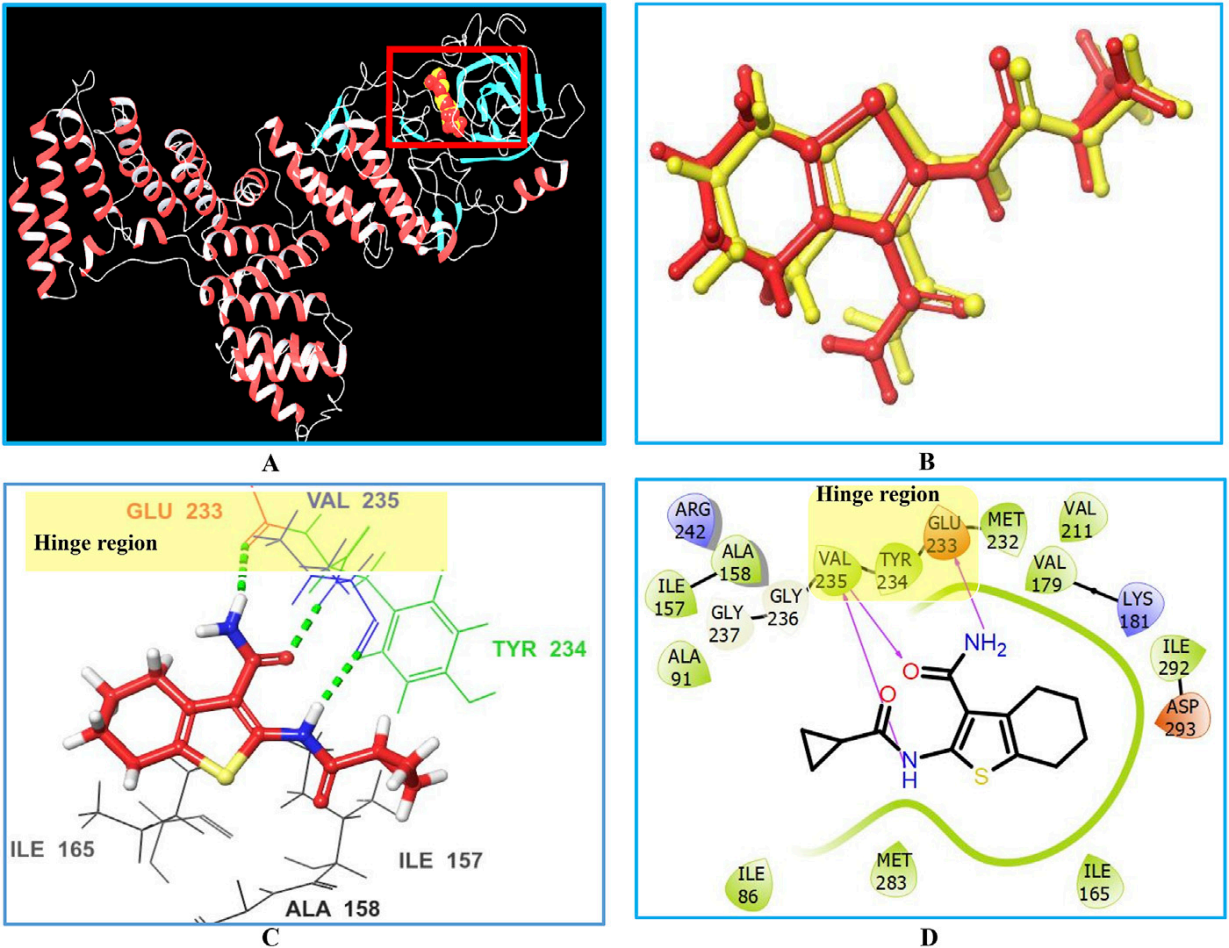
Interactions between the co-crystallized ligand (AX20017) and PknG (PDB ID: 2PZI) as the target enzyme. Panel **(A)** displays the 3D crystal structure of the target enzyme complexed with the native AX20017 ligand, Panel **(B)** presents a superposition of the co-crystallized ligand conformation (yellow) and the docked ligand conformation (red), with an RMSD value of 0.28 Å. Panel **(C)** shows the corresponding 3D crystal structure. The interacting amino acid residues at the binding site are represented by their three-letter codes. Hydrogen bond interactions are indicated by dotted green lines in panel **(C)** and solid magenta lines in panel **(D)**.

**FIGURE 5:**
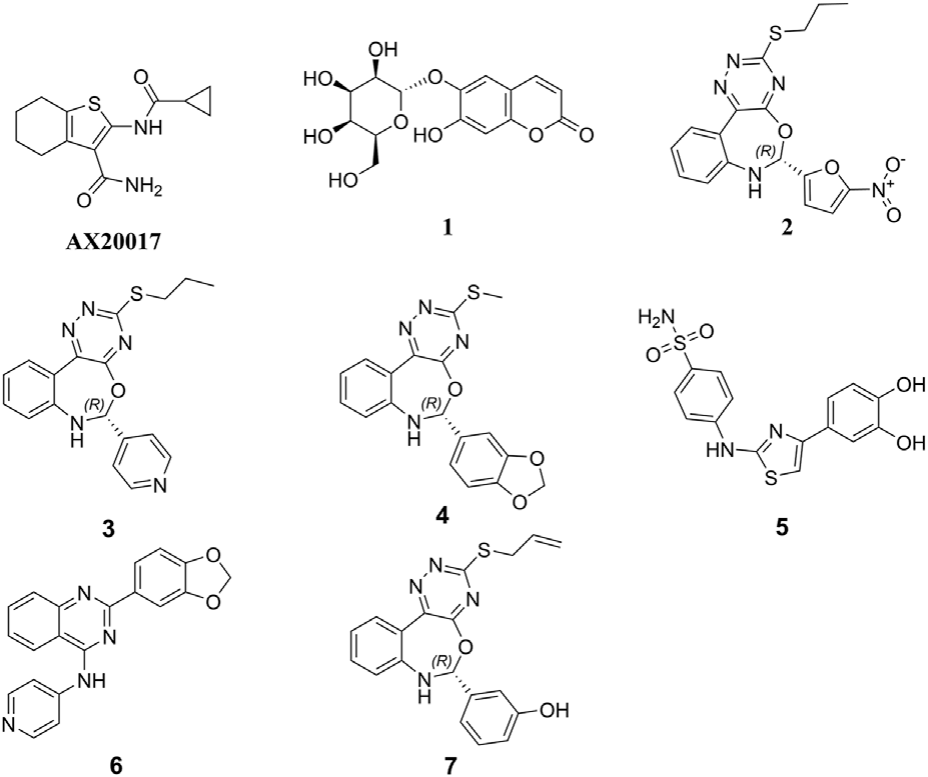
Chemical structures of the top 7 hits and the co-crystal ligand AX20017.

Initially, the 3D crystal structure of PknG, of M.tb (PDB ID 2PZI) in complex with tetrahydrobenzothiophene (AX20017), was downloaded from the protein data bank (PDB: https://www.rcsb.org/) for structure-based virtual screening. The bound AX20017 (Figure 4A) was docked into the same binding cavity to confirm the validity of the docking protocol and the RMSD between the docked and the experimental co-crystallized poses (Figure 4B) was 0.28 Å which was within the acceptable range (Santiago-Silva et al., 2023). As shown in Figures 4C,D, AX20017 formed three H-bonds with the hinge region residues using its amide side chains, two with Val235 and the other with Glu233. Further, hydrophobic interactions were observed with residues Ile86, Asp87, Ala91, Ile157, Ala158, Ile165, Val179, Tyr234, Met283, and Ile292. These results were found consistent with what has been reported by Arica-Sosa et al. (2022). Following this step, a multilevel docking approach was employed, beginning with docking in Glide High Throughput Virtual Screening mode (Glide-HTVS), followed by Glide Standard Precision mode (Glide-SP), and concluding with Glide Extra Precision mode (Glide-XP). Molecules exhibiting a Glide-XP docking score lower than that of AX20017 (−8.14 kcal/mol) were then selected as top hits Chemical structures of these Hits **1-7** are provided in Figure 5. The analysis of docking results indicated that Hits **1-7** displayed XP docking score within the range of −8.31 to −12.75 kcal/mol (Table 1). Hit **1**, a chromene glycoside, had the highest docking score (−12.75 kcal/mol) followed by the oxazepine derivative **7** which scored −10.03 kcal/mol. The remaining top hits generally exhibited similar docking scores, all of which were higher than that of the reference ligand (Table 1). While Hits **2, 3, 4**, and **7** feature the tricyclic triazinobenzoxazepin ring system, Hits **5** and **6** are derivatives of benzene sulphonamide and quinazoline, respectively.

**TABLE 1:** Docking results and the interaction forces of the top hit molecules.

PubChem ID	Hit	Docking score (kcal/mol)	Interactions	
			H-bond	Pi-cation
673,481	Co-crystallized ligand(AX20017)	‒8.14	Val235 and Glu233	-
6,604,620	**1**	‒12.75	Val235; Tyr234; Glu233; Gly237; Ser239 and Arg242	-
285,8302	**2**	‒8.96	Val235 and Arg242	-
285,8937	**3**	‒8.31	Val235	Arg242
2,849,297	**4**	‒8.53	Val235 and Arg242	-
2,928,782	**5**	‒8.76	Ala91; Ile157; Lys181; Val235; Arg242 and Glu280	-
3,234,587	**6**	‒8.86	Val235 and Lys241	-
6,410,292	**7**	‒10.03	Val235 and Ile157	-

**FIGURE 6:**
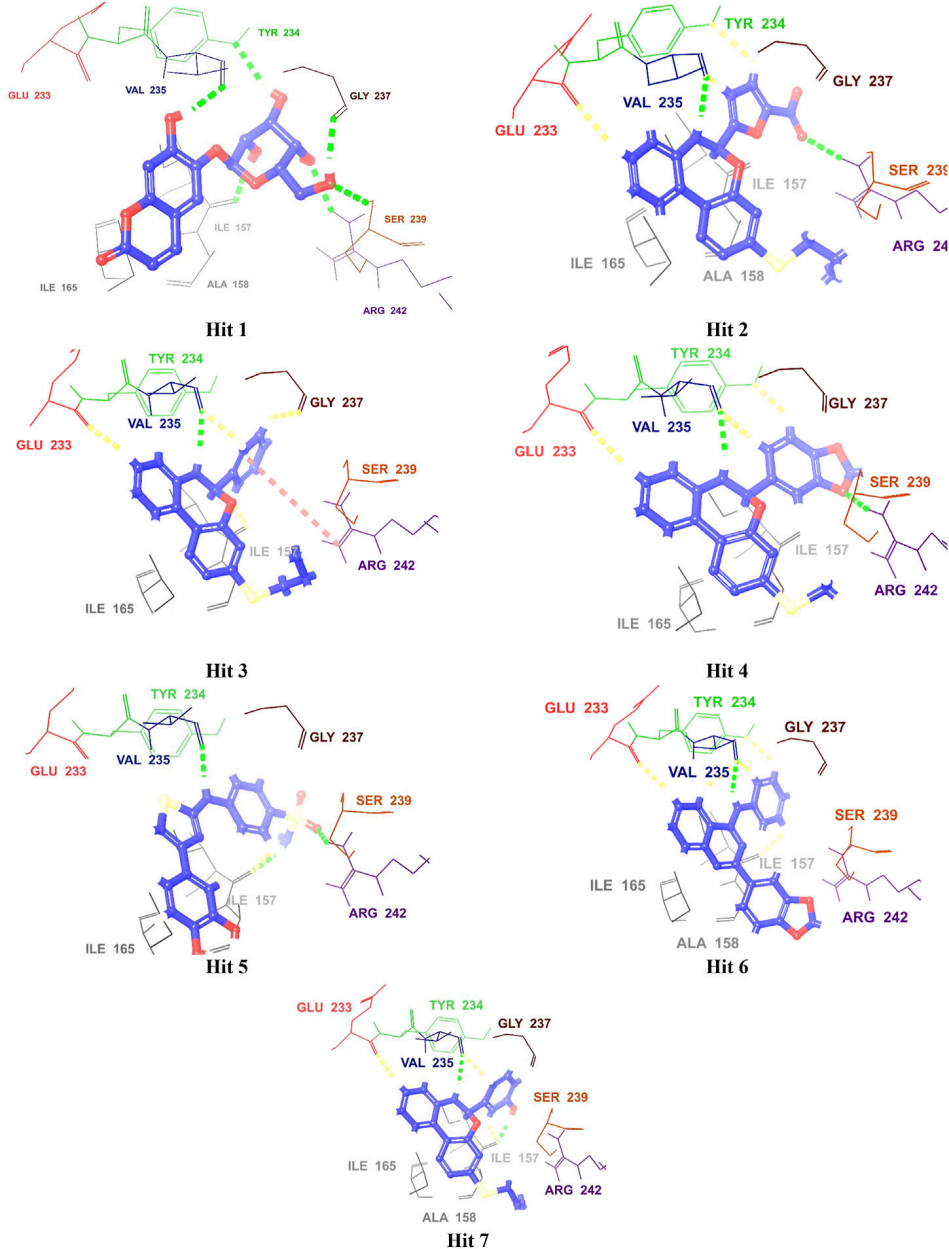
3D Interaction diagrams of top identified Hits (1–7) with the M. tb PknG binding site. Hydrogen bonds (green), aromatic-H bonds (yellow), and π-cation interactions (red) shown. Amino acid residues are displayed using three-letter codes.

In addition, Hits **1‒7** formed multiple hydrogen bonds with various residues in the catalytic site (Figure 6; Supplementary Figure S1), exhibiting interaction patterns similar to those reported for experimentally validated molecules (Singh et al., 2015; Arica-Sosa et al., 2022).

**FIGURE 7:**
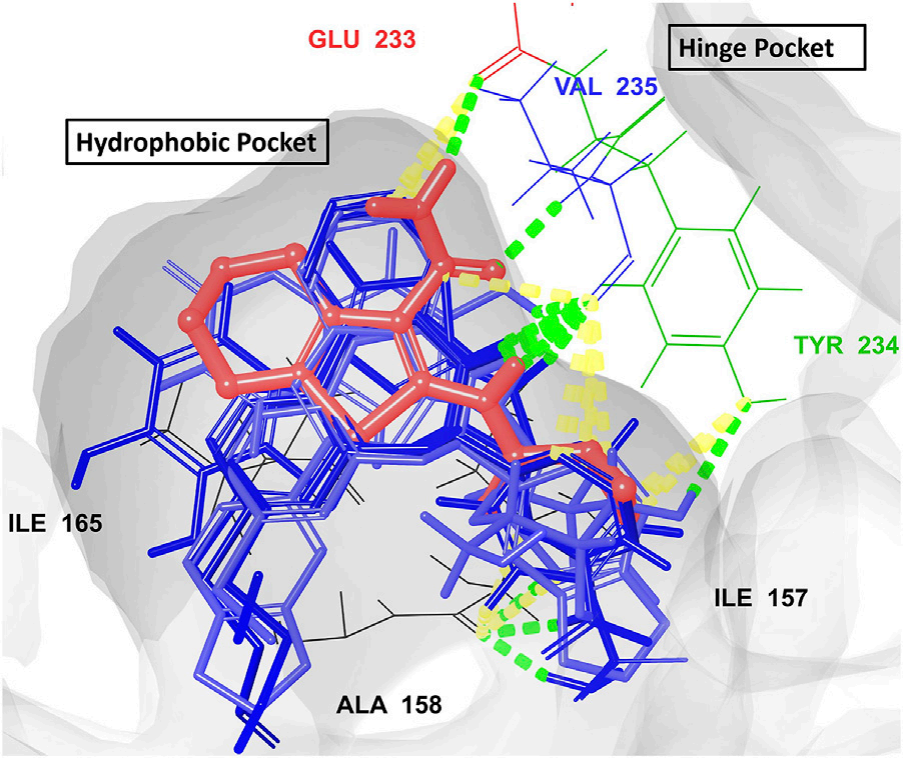
The overlay of the co-crystallized ligand AX20017 (red) and the docked conformations of the top 7 hits (blue) interacting with the 2PZI binding pockets (the hydrophobic and the hinge). The conventional H-bonds are shown in green color, while the aromatic H-bonds are shown in yellow. The most critical residues for proper interaction and for ligand-enzyme complex stability (Glu233 and Val235) are shown in red and blue colors, respectively.

All hits were positioned precisely within the same binding site as the reference compound AX20017, which validated the robustness of the docking protocol and confirmed the hits’ suitability as potential inhibitors. Detailed analysis of interaction patterns revealed that the hits formed key hydrogen bonds with **Val235**, a critical residue located in the hinge region. This interaction was particularly significant because the hinge region played a central role in maintaining the structural integrity and proper folding of the active site, thereby facilitating effective ligand binding. Hydrogen bonding with Val235 was pivotal for stabilizing the ligand-enzyme complex, as previously reported by Arica-Sosa et al. (2022). This residue, due to its position in the hinge region, mediated essential interactions that anchored the ligand within the binding pocket. Furthermore, similar to AX20017, the hits engaged in hydrophobic interactions and π-π stacking with neighboring residues, which further enhanced the binding affinity. Notably, the spatial positioning and interaction patterns observed in Figures 6, 7 confirmed that the hits not only mimicked the binding mechanism of AX20017 but also exhibited comparable interaction strength, reinforcing their potential as strong inhibitors. These findings underscored the importance of targeting the hinge region to achieve high binding stability and effective inhibition of the enzyme. By exploiting such conserved interactions, these hits provided a promising starting point for further optimization and lead development.

### 3.2 Binding free energy calculations

To estimate the binding free energy of the enzyme-ligand complexes, MM-GBSA method was utilized. This is one of the most common methods used to estimate the binding free energy of the small molecules to their respective macromolecular targets (Wang et al., 2019). This could be attributed to its relatively high scoring function accuracy relative to the molecular docking simulation and is thus employed here to improve the results of virtual screening (Genheden and Ryde, 2015). Table 2 displays the binding free energy values for the top 7 hits alongside that of the co-crystal ligand. Hits **1‒7** displayed free binding energies ranging from −60.92 to −69.95 kcal/mol, which were lower than that of the co-crystal ligand (−60.23 kcal/mol), pointing to their higher affinity towards the enzyme binding pocket.

**TABLE 2:** Energy terms contributing to the free energy (Gbind) of Hits 1-7 and the co-crystal ligand AX20017.

Hit	MMGBSA dG bind (kcal/mol)	MMGBSA dG bind coulomb	MMGBSA dG bind Hbond	MMGBSA dG bind lipo	MMGBSA dG bind packing	MMGBSA dG bind vdW	MMGBSA dG bind covalent	MMGBSA dG bind solv GB
Co-crystal ligand	‒60.23	‒20.65	‒1.51	‒19.54	0.00	‒44.71	0.41	25.76
**1**	‒61.27	‒38.16	‒3.63	‒20.85	0.00	‒41.03	7.07	35.32
**2**	‒64.55	‒0.221	‒1.36	‒25.21	‒0.04	‒52.91	0.41	23.78
**3**	‒69.95	‒60.75	‒0.52	‒27.80	‒0.14	‒49.80	0.95	68.10
**4**	‒60.92	‒6.58	‒1.02	‒27.54	‒0.01	‒54.27	1.41	27.09
**5**	‒60.98	‒36.32	‒4.08	‒19.32	‒0.13	‒43.44	4.07	38.23
**6**	‒69.14	‒56.21	‒1.08	‒22.95	‒0.32	‒50.44	1.55	60.31
**7**	‒65.81	‒9.95	‒1.04	‒27.40	‒0.21	‒52.33	1.25	23.86

**FIGURE 8:**
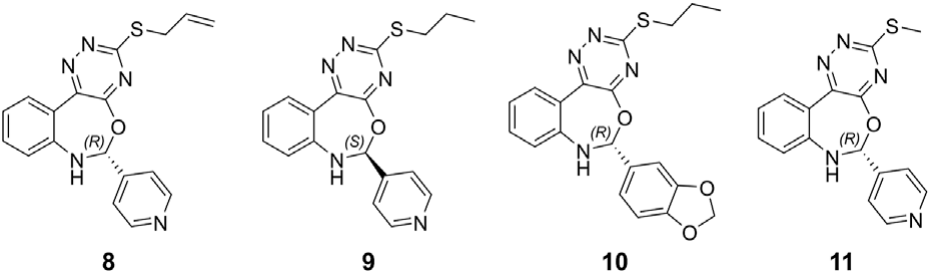
Chemical structures of the designed triazinobenzoxazepin derivative 8‒11.

Hits **3**, the triazinobenzoxazepin derivative, showed the lowest free binding energy (−69.95 kcal/mol). It is evident that out of the top 7 hits, 4 hits incorporate the triazinobenzoxazepin ring system. Replacement of the nitrofuran moiety attached to the C-2 of benzoxazepine ring in **2** by the pyridine ring in **3** increased the binding affinity. Unsaturation of the C2-C3 of the propyl side chain in **3** (compound **8** shown in Figure 8) resulted in slight reduction in the binding affinity (−68.99 kcal/mol). Inversion of the chirality in Hit **3** (compound **9** shown in Figure 8) increased both the docking score (−5.28 kcal/mol) and the total binding energy (−65.88 kcal/mol) indicating the importance of the *R* configuration for higher affinity. Substitution of the pyridine ring in compound **8** with the hyroxyphenyl ring afforded Hit **7** with relatively lower binding affinity (−65.81 kcal/mol). Extension of the methyl group in **4** to n-propyl as in **2** and **3** (compound **10** shown in Figure 8) reduced the binding affinity (−58.17 kcal/mol), as did the shortening of the n-propyl in Hit **3** to methyl in compound **11** (Figure 8) (binding energy = −69.47 kcal/mol). Thus, it could reasonably be concluded that, pyridine ring linked to C-2 of the benzoxazepine ring together with the n-propyl chain attached to the triazine ring might be important for optimal affinity.

**FIGURE 9:**
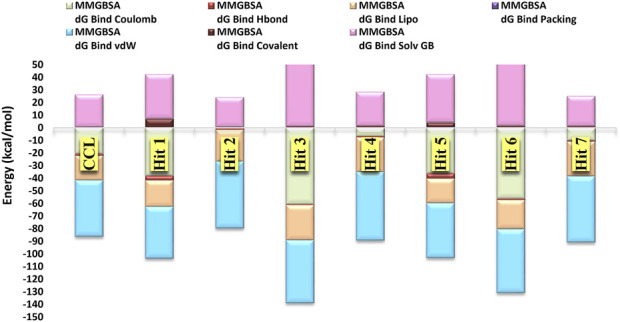
The major energy components contributing to the free energies (∆Gbind) of the top 7 hits and the co-crystal ligand, including electrostatic (∆Gbind Coulomb), Van der Waals (∆Gbind VDW), lipophilic (∆Gbind Lipo), and hydrogen bonding (∆Gbind Hbond).

The overall binding free energy for each hit arose from multiple energy components (Table 2; Figure 9), encompassing Coulombic energy (Coulomb), covalent bonding (Covalent), hydrogen bonding (Hbond), lipophilic interactions (Lipo), π-π packing interactions (Packing), generalized solvent binding (Solv GB), and van der Waals interactions (VdW)Among these terms, the contribution of the VdW energy was more than the others in the total binding energies of the top 7 hits and the co-crystal ligand. Coulomb energy came in the second place after the VdW energy as contributing force of interaction for Hits **1**, **3**, **5** and **6** alongside the co-crystal ligand. In contrast, for the triazinobenzoxazepin **2**, **4** and **7** the second most contributing force was the lipophilic one. Interestingly, the triazinobenzoxazepin **3** (the hit with the highest affinity), coulomb energy was the principal contributing force of interactions. This could be attributed to the presence of the ionizable positively charged pyridine ring, which interacts via pi-cation forces with Arg242. As for co-crystal ligand, the contributions of H-bonding and the π-π packing forces to the total binding energy of the top 7 hits were very little. Overall, the outcome of this analysis indicates that, Hits **1‒7** possessed affinity towards the PknG binding domain higher than that of the co-crystal ligand. Additionally, the decomposed energy terms contributing to the total free binding energies for Hits **1‒7** were similar to those of the co-crystal ligand and were therefore considered all for the next ADMET filtering step.

**FIGURE 10:**
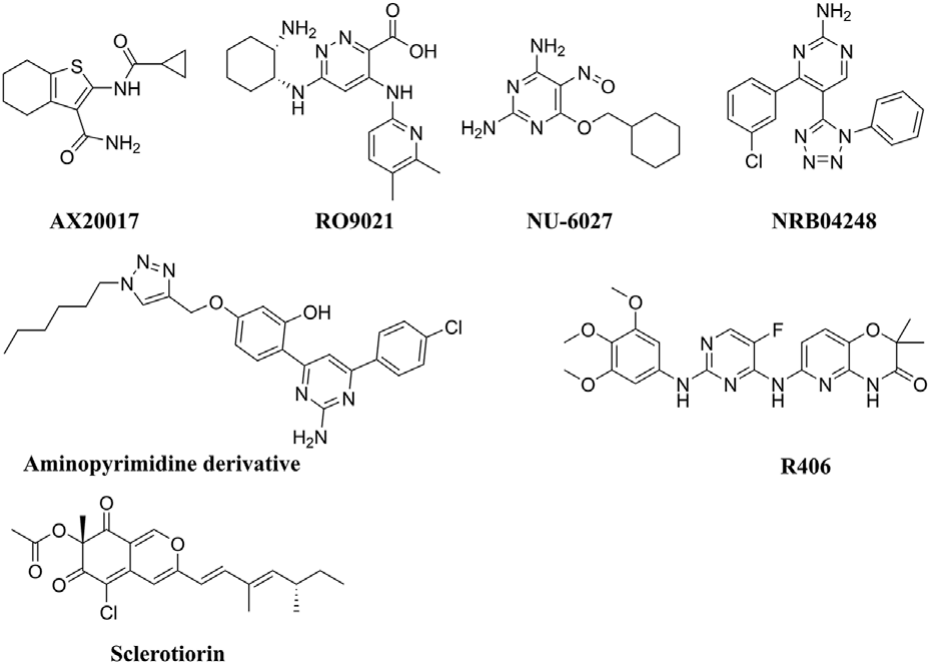
Chemical structures of the experimentally validated M.tb PknG inhibitors.

To provide context for our findings, we conducted a comparative analysis with previously reported potent inhibitors of M.tb PknG (Figure 10). Tables 1–3 revealed notable differences in docking scores and binding energies for M.tb PknG inhibitors. Tables 1 and 2 summarized the results for the computationally identified Hits **1–7**, while Table 3 provided data for experimentally validated inhibitors. The docking scores of Hits **1–7** were significantly better than those of the validated inhibitors. For example, Hit **1** achieved a docking score of −12.75 kcal/mol, outperforming AX20017, the co-crystallized ligand in Table 3, which had a docking score of −8.14 kcal/mol. Other hits, such as Hit **7** (−10.03 kcal/mol) and Hit **6** (−8.86 kcal/mol), also showed stronger docking affinities compared to most validated inhibitors, whose scores ranged from −2.43 kcal/mol to −8.14 kcal/mol. In terms of binding energies, the Hits **1–7** in Table 2 demonstrated more favorable values. Hit **3** exhibited the strongest binding energy at −69.95 kcal/mol, followed by Hit **6** (−69.14 kcal/mol) and Hit **7** (−65.81 kcal/mol). In contrast, AX20017, the most potent validated inhibitor in Table 3, had a binding energy of −60.23 kcal/mol. Other inhibitors in Table 3 showed weaker binding energies, ranging from −32.23 kcal/mol to −60.23 kcal/mol. Overall, the results indicated that Hits **1–7** exhibited stronger predicted binding affinities and docking scores compared to the experimentally validated inhibitors. These findings suggest that the computationally identified hits have promising potential as superior candidates for M.tb PknG inhibition, though experimental validation would be necessary to confirm their effectiveness. As shown in Supplementary Figure S2, with the exception of AX20017 and RO9021, the experimentally validated inhibitors displayed less efficient interactions compared to our identified hits (Supplementary Figure S1), particularly in terms of binding to the hinge region. This difference in interaction patterns correlates with the lower binding affinities observed for these inhibitors, as their suboptimal binding to critical residues in the hinge region likely reduces the overall stability and strength of the ligand-enzyme complex.

**TABLE 3:** Experimentally validated M.tb PknG inhibitors with docking scores, binding free energies, and IC₅₀ values.

No.	Name	Docking score (kcal/mol)	Binding energy (kcal/mol)	IC_50_	References
**1**	AX20017 (Co-crystallized ligand)	‒8.14	‒60.23	0.2 ± 0.04 μM	Scherr et al. (2007), Arica-Sosa et al. (2022)
**2**	RO9021	‒2.43	‒52.48	4.4 ± 1.1 μM	Arica-Sosa et al. (2022)
**3**	NRB04248	‒4.75	‒38.17	43% inhibition at 25 μM	Singh et al. (2015)
**4**	Aminopyrimidine derivative	‒4.48	‒42.49	43 ± 2.94 at 100 μM	Anand et al. (2012)
**5**	NU-6027	‒4.83	‒32.23	at 50 μM selectively inhibits PknG	Kidwai et al. (2019)
**6**	R406	‒5.37	‒47.53	83.8% inhibition at 16.1 µM	Kanehiro et al. (2018)
**7**	Sclerotiorin	‒1.68	‒24.43	76.5 μM	Chen et al. (2017)

Compared to the reported PknG inhibitors, the identified hits in this study represent novel classes of PknG inhibitors, marking a significant advancement in tuberculosis drug discovery. To the best of our knowledge, these are the first reported examples of PknG inhibition for the following scaffolds: chromene glycoside, triazinobenzoxazepin derivative, quinzoline, and benzene sulphonamide. These scaffolds are not only structurally unique but also provide a promising basis for the development of new anti-tubercular therapies targeting the PknG enzyme, which is essential for the survival and pathogenicity of M.tb. The chromene glycoside scaffold, while known for its diverse biological activities (Amen et al., 2021), has never been explored for PknG inhibition before. Similarly, the triazinobenzoxazepin derivatives, which have been previously studied for their activity in other therapeutic areas (Stefaniak and Olszewska, 2021), are also reported here for the first time as potential PknG inhibitors. The quinzoline scaffold, traditionally associated with anti-tubercular properties (Kushwaha et al., 2023), has been linked to PknG inhibition in this study for the first time. This discovery is particularly exciting, as quinzoline derivatives have shown promise in other aspects of tuberculosis treatment, and their application as PknG inhibitors may offer an additional, synergistic mechanism of action. Finally, the benzene sulphonamide scaffold, widely known for its antimicrobial properties (Kapısuz et al., 2024), has not previously been explored in the context of PknG inhibition. The identification of this scaffold as a potent PknG inhibitor is a significant milestone and could lead to the development of compounds that specifically target drug-resistant strains of M.tb, a growing concern in the fight against tuberculosis. Collectively, these scaffolds represent a promising foundation for the development of new anti-TB therapies. The discovery of these novel scaffolds as PknG inhibitors could therefore pave the way for more effective treatments, particularly in the context of drug resistance, marking a significant advancement in the search for new tuberculosis therapies. Current anti-TB drugs face significant limitations, including lengthy treatment durations, low cure rates ranging from 40% to 50%, and the challenge of being confounded by factors such as poor nutrition and co-infections. Additionally, these drugs often require large dosages and are associated with more side effects due to poor pharmacokinetic properties (Wei et al., 2024). These shortcomings present a critical mechanistic gap in existing treatments. The identified hits in this study address this gap by specifically targeting PknG, offering a novel approach to inhibit M.tb persistence and survival within the host. These hits are unique in that they have the potential to target non-proliferating mycobacteria during the latency stage and prevent the development of Mtb-resistant strains, which could significantly enhance treatment outcomes. Furthermore, these hits have the potential to be used in combination with existing anti-TB drugs, increasing their efficacy, reducing the risk of resistance, and overcoming the limitations of current therapies. By targeting a novel pathway and providing an additional layer of treatment, these inhibitors could not only improve therapeutic efficacy but also shorten treatment durations, offering a crucial solution to address the global TB crisis (Farhat et al., 2024).

However, off-target effects in PknG inhibitors pose a significant challenge in drug development, as these unintended interactions with non-target molecules can lead to adverse biological responses. Such interactions compromise treatment specificity and may cause harmful side effects, limiting the clinical utility and safety of these compounds. Given that PknG is a kinase in M.tb, understanding kinase off-target effects is critical. Numerous studies have shown that off-target kinase inhibition can result in adverse outcomes, highlighting the necessity of comprehensive screening to ensure the selectivity of the identified hits as PknG specific inhibitors and reduce potential therapeutic risks (Green et al., 2023). Our docking analysis of the top identified hits revealed interactions with residues in the PknG binding site that are specific to PknG, such as Ile165, Val179, Gly236, Ile292, Ile87, and Ala92. These residues are absent in the 11 other serine/threonine protein kinases (STPKs) from M.tb and are also absent in human kinases, which aligns with the behavior of the previously reported inhibitor NU-6027 (Kidwai et al., 2019). Furthermore, the frequencies of occurrence for the residues Ile165, Val179, Gly236, and Ile292 in the surrounding PknG binding site are minimal (Scherr et al., 2007). The combination of these residues is not observed in any other human kinase sequences. Additionally, the Ile87 and Ala92 residues, which are located within the amino-terminal peptide stretch, are also unique to the ligand-binding pocket of PknG (Scherr et al., 2007; Kidwai et al., 2019). While these findings suggest that our identified hit may exhibit selectivity, it is important to note that these conclusions are based solely on *in silico* studies and require further experimental validation. Such validation includes conducting comprehensive kinase profiling to identify potential off-target interactions and chemically modifying the inhibitors to enhance selectivity for PknG. Structural modifications can be made to prevent binding to off-target kinases while preserving strong affinity for PknG.

### 3.3 *In silico* ADMET profiling

*In-silico* prediction approaches of ADMET properties for potential hits have become an integral component of modern drug discovery process (Vrbanac and Slauter, 2017; Ferreira and Andricopulo, 2019). Owing to their reduced cost compared to the experimental ones, these approaches have attracted the attention of the scientific community (Ntie-Kang, 2013). In this context, *in silico* ADMET prediction of the 7 top-ranked hits was achieved using the QikProp module in Schrödinger software and the publicly accessible webserver ADMETLab 2.0. The predicted descriptors were compared with the limiting ranges. In general, results indicated positive physicochemical and pharmacokinetic properties for the 7 top ranked hits (Supplementary Table S1). The overall ADME-compliance score–drug-likeness parameter (indicated by #stars) of these hits were within the specified limit indicating that they had properties similar to those for 95% of known drugs. In addition they showed no violations to Lipinski (Rule of Five) or Rule of Three. With Hits **1** and **5** showing medium oral absorption (<60%), the rest exhibited high oral absorption rate (>85%) pointing for their potential for oral administration, a favorable criterion for ideal anti-TB drug (Sotgiu et al., 2015). Since TB could involve CNS as a primary site of infection (Rock et al., 2008) anti-TB drug’s crossing of the BBB is considered an important feature. To this end, the predicted brain/blood partition coefficient (QP log BB) and the Polar Surface area (PSA) are Qikprop descriptors employed to evaluate the BBB permeability of potential drug candidates (Dighe et al., 2020). The values for these descriptors for the top-ranked hits were within the designated ranges, indicating their high potential for CNS penetration. Further, the efficient distribution of Hits **1‒7** in the human body was estimated using apparent Caco-2 (QPPCaco) and MDCK cell (QPPMDCK) permeability parameters (Dighe et al., 2020; Amengor et al., 2022). Excluding the chromene glycoside, Hit **1**, the rest displayed an enhanced permeability in both Caco-2 and MDCK cells (the predicted values fell within the recommended ranges) confirming their potentiality for efficient distribution in the human body following oral administration. Binding of a given drug to the blood plasma proteins is a crucial factor affecting the efficacy of that drug, since it determines its free concentration that would be available to cross the biological membranes and consequently to interact with its target (Hann et al., 2022). Accordingly, the QPlogKhsa parameter was done here for evaluating the binding of Hits **1‒7** to the human albumin which is considered the highly abundant plasma protein capable of binding to a variety of drugs (Hann et al., 2022). Hits **1-7** were found to be compliant to this parameter, and thus would be expected to circulate in the blood smoothly from side to side until they reach their site of action. Human Ether-a-go-go Related Gene (HERG) encodes a potassium ion channel that is documented to play a key role in a fatal type of arrhythmia known as torsade *de pointes* (Lamothe et al., 2016). Further, it represents a macromolecular via which several drugs mediate their cardiotoxic effects (Lamothe et al., 2016; Amengor et al., 2022). In this context, the IC_50_ value for blockage of HERG K^+^ channels parameter (QPlogHERG) was used in the present study to evaluate the propensity of Hits **1‒7** to impose cardiotoxic side effects. All the investigated hits, except Hit **1**, were detected to have the potential to induce HERG-related cardiotoxic effects since their predicted IC_50_ values for blockage of HERG K^+^ channels fell outside the specified limit (concern below −5). The number of likely metabolic reactions of a drug candidate is an essential descriptor used to identify whether it could reach its site of action following entering the blood circulation. The computed numbers of possible metabolic reactions for Hits **1‒7** were between 2‒5 which were within the recommended set limit (1–8). Overall, attractive pharmacokinetic profiles. Given the above information, Hits **1‒7**, in general, had appropriate ADMET profiles, nevertheless further optimization is required to boost up their therapeutic properties. Based on the obtained favorable ADME profiles for Hits **1‒7**, the research was directed to assess their potential toxicity. *In-silico* assessment of potential adverse effects of a drug candidate has now become a routine practice in the pharmaceutical industry, particularly at the earlies stages of the drug discovery program to reduce the drug attrition rate (Liu et al., 2023; Rababi and Nag, 2023). In the same regard, the webserver ADMETLab 2.0 was used to perform toxicity evaluation of Hits **1–7**. As shown in Supplementary Table S2, all of the investigated Hits had the high risk to induce liver injury. Hits **1** and **5** had the low probability to be hepatotoxic (the output values < 0.3), Hit **7** had the borderline low probability to precipitate liver toxicity (the output value < 0.7), the rest had greater potential to be hepatotoxic (the output values > 0.7). It was noticed that the triazinobenzoxazepines **2‒4** and **7** showed active remarks in both AMES and respiratory toxicity parameters (output values > 0.7). Estimation of the carcinogenic properties of the investigated hits pointed to the high risk of Hits **2** and **4** to induce cancer. To get an insight on the possible side effects that could emerge upon prolonged and frequent administration of Hits **1‒7**, rat oral acute toxicity descriptors (ROA) were determined. It was observed that all of the hits were safe and having less potential to impose acute toxic effects in human. Calculated values for eye corrosion (EC), eye irritation (EI) and skin sensitization (SkinSen) were found to meet the standards for safe drug candidate. In addition, we conducted a comparative study between the most widely used and clinically established first-line anti-TB drug Isoniazid (INH) and Hit **1** to gain a deeper understanding of their pharmacokinetic characteristics and toxicity profiles (Supplementary Tables S1, S2). The anti-TB drug INH had a smaller molecular weight (137.14) and solvent accessible surface area (SASA: 329.65) compared to Hit **1**, indicating that INH was smaller in size and had different polarity. INH demonstrated superior permeability (QPPCaco: 273.87 nm/sec) and solubility (QPlogS: –1.03) than Hit **1**, suggesting better drug-likeness. Additionally, INH had fewer hydrogen bond acceptors (4.5) and higher human oral absorption (66.7%) compared to Hit 1 (42.33%). Both INH and Hit **1** complied with Lipinski’s Rule of Five, although Hit **1** violated Jorgensen’s Rule of Three. Regarding toxicity, INH displayed a poorer profile for hepatotoxicity (H-HT: 0.71) and drug-induced liver injury (DILI: 0.70) compared to Hit **1** (0.12 and 0.81, respectively). INH also showed higher mutagenicity (AMES: 0.93) and skin sensitization (SkinSen: 0.98) compared to Hit **1** (0.33 and 0.69). However, Hit **1** exhibited significantly better respiratory toxicity (0.03) than INH (0.99). Both compounds had low recommended daily doses (FDAMDD: 0.06 for INH, 0.0 for Hit **1**), and although INH had a higher eye irritation potential (EI: 1.00), Hit **1** showed a more favorable value (0.23) for this parameter. In summary, while INH demonstrated better pharmacokinetics, Hit **1** exhibited a safer toxicity profile, making it a promising candidate with potential advantages in terms of safety. Thus, the selection of Hit **1** was primarily driven by its superior safety profile, which emerged as the most significant factor distinguishing it from the other top hits. Among the evaluated candidates, Hit **1** demonstrated the lowest predicted toxicity risks, ensuring its suitability for further development. In addition, it exhibited favorable ADMET properties, which further supported its drug-likeness and potential for clinical translation. The prioritization of Hit **1** was also influenced by its balanced physicochemical characteristics, aligning with the criteria for successful drug candidates. This comprehensive evaluation provided strong justification for advancing Hit **1** to more detailed computational studies. These investigations aim to explore its structural and electronic features, laying the groundwork for targeted modifications to enhance its efficacy, selectivity, and overall therapeutic potential.

### 3.4 Quantum computational calculations

**FIGURE 11:**
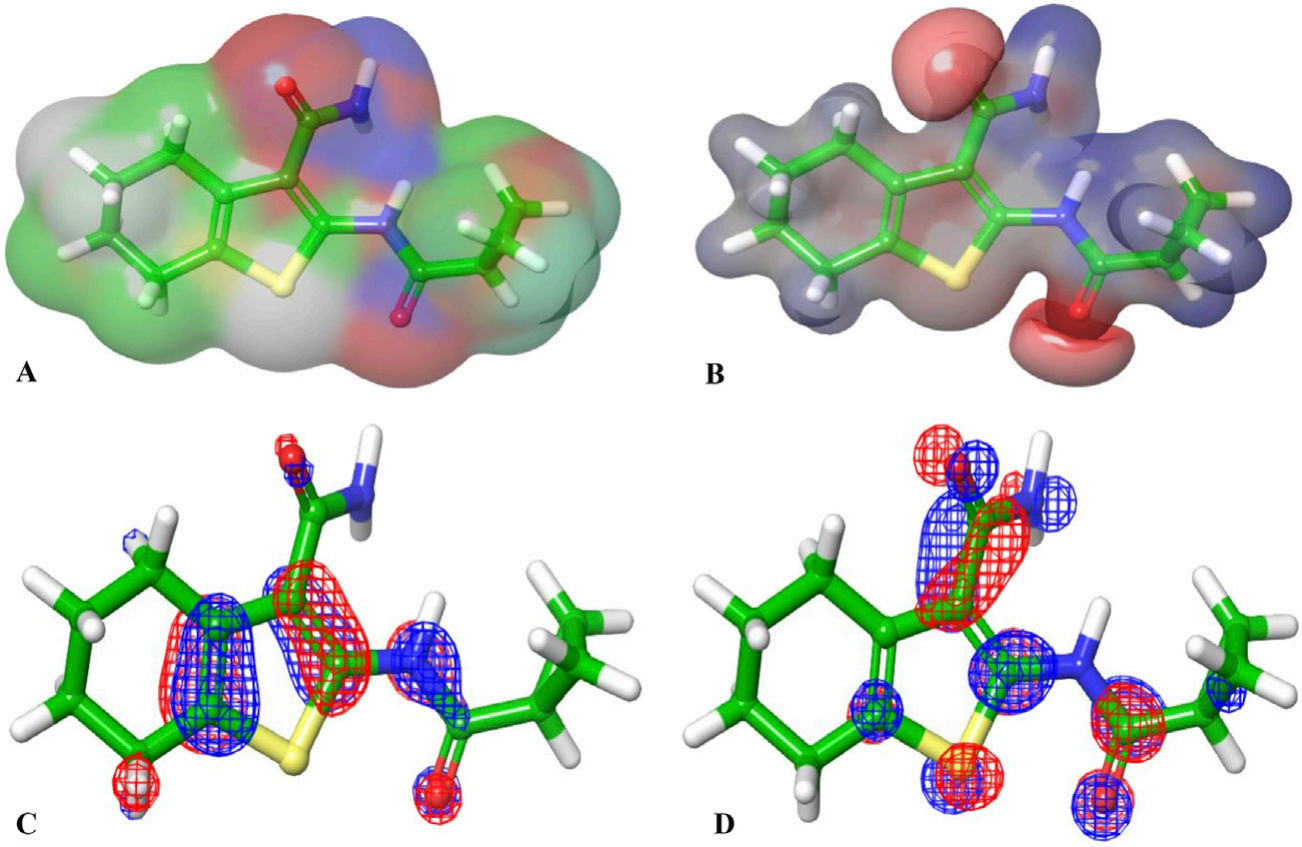
**(A, B)** are the electron density and MESP of the reference ligand; **(C, D)** are the molecular orbital distribution plots of E_HOMO_ and E_LUMO_ of the reference ligand, respectively.

The molecular orbital and electronic features of the investigational hits are commonly computed employing quantum mechanical methods (Zhou et al., 2010). In this study the DFT (density functional theory) analysis of Hit **1** and the co-crystal ligand was performed using Jaguar module of Schrödinger suite. Table 4 illustrates the Frontier Molecular orbitals (FMOs) E_HOMO_ and E_LUMO_, the HOMO-LUMO Gap (HLG) along with the quantum chemical reactivity descriptors of Hit **1** and the co-crystal ligand. Molecule’s electron affinity is directly related to its E_LUMO_, while its ionization potential is associated with its E_HOMO_ (Ahmad et al., 2023). Hit **1** exhibited E_HOMO_ value similar to that of the co-crystal ligand indicating their similar ability to donate electrons to an acceptor molecule, meanwhile, it displayed more negative E_LUMO_ pointing to its relatively higher electron affinity as compared to the co-crystal ligand. The E_HOMO_ and E_LUMO_ plots of the co-crystal ligand (Figures 11C,D) implies that nearly the entire structural units of the molecule involved in FMOs and are thus could significantly participate in the electron donation/acceptance processes.

**TABLE 4:** Quantum chemical reactivity descriptors of Hit 1 and the co-crystal ligand (eV).

Compound	E_HOMO_ (eV)	E_LUMO_ (eV)	HLG	Electron affinity	Ionization potential	Chemical hardness	Chemical softness	Electronegativity	Global electrophilicity index
Co-crystal ligand	‒5.67	‒1.10	4.57	1.10	5.67	2.28	0.43	3.38	2.50
Hit 1	‒6.10	‒1.85	4.25	1.85	6.10	2.12	0.47	3.97	3.71

**FIGURE 12:**
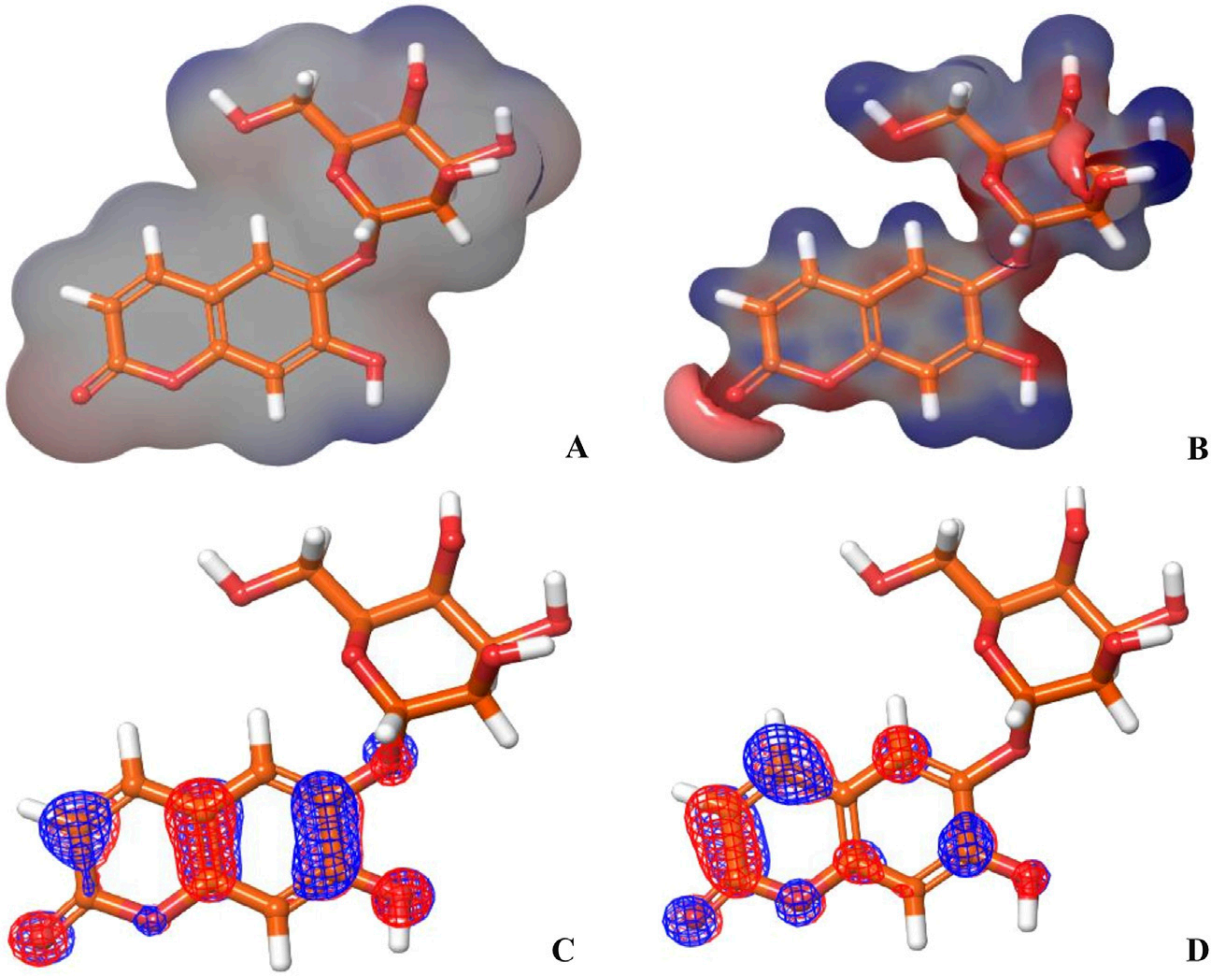
**(A, B)** show the electron density and MESP of Hit 1; **(C, D)** show the molecular orbital distribution plots of E_HOMO_ and E_LUMO_ of Hit 1, respectively.

On the other hand, the E_HOMO_ and E_LUMO_ plots of Hit **1** (Figures 12C,D) localized exclusively over the chromene ring rather than the sugar moiety, HLG indicates whether the molecule is kinetically and chemically stable or not (Alsehli et al., 2021). Both, the co-crystal ligand and Hit **1** demonstrated similar HLG values (Table 4) implying that they had similar chemical and kinetic stability and are thus having similar electrons transferring and exchanging liabilities. Likewise, the molecular hardness (ƞ) and softness (σ) of the co-crystal ligand and Hit **1** were similar which further confirmed their intermediate chemical stability since these descriptors are directly related to HLG. It was observed that the computed electronegativity (χ) for the co-crystal ligand and Hit **1** were 3.385 and 3.975 eV, respectively, indicating the comparatively high potential of Hit **1** for electron attraction. Further, the global electrophilicity index (ω) was calculated to evaluate the capacity of Hit **1** and the co-crystal ligand to accept from molecules. As shown in Table 4, the calculated electronegativity ω of Hit **1** and the co-crystal ligand is found to be 2.507 and 3.71, eV, respectively which reflects the strong electrophilic property of the identified Hit relative to that of the reference ligand (Rana et al., 2021; Burkhanova et al., 2022). The electron density and the MESP of the co-crystal ligand and Hit **1** (Figures 11A, B, 12A, B, respectively) were computed to display the electron-rich, electron-deficient and neutral regions of these molecules. In addition it also provide information regarding the molecular size and shape of the tested compounds. The electrostatic potential differences on the regions are depicted in red, yellow, and blue colors which refer to the highly negative, negative and the highly positive molecular regions, respectively. While the positive regions represent the favorable site for nucleophilic attack, the negative regions are the favorable site for electrophilic attacks. As illustrated in Figures 11A, B, 12A, B, the negative surfaces are presented in the electronegative oxygen atoms alongside the C=C bonds, meanwhile the positive ones are localized at the hydrogen atoms. These electrostatic surfaces were shown to play a critical role in helping co-crystal ligand and Hit **1** to interact with diverse forces of interactions to the PknG catalytic site.

### 3.5 MD simulation

**FIGURE 13:**
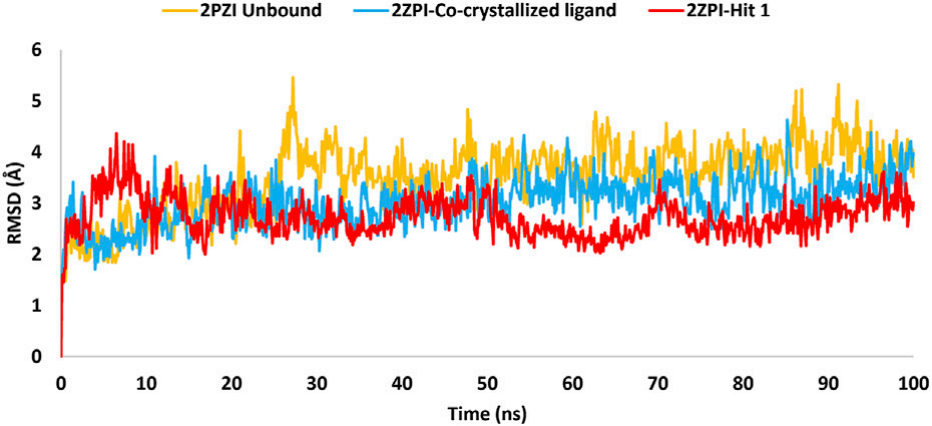
RMSD plot generated through MD trajectories of the C-α atoms of the unbound PknG (PDB: 2PZI) and its complexes with co-crystallized ligand, along with Hit 1.

**FIGURE 14:**
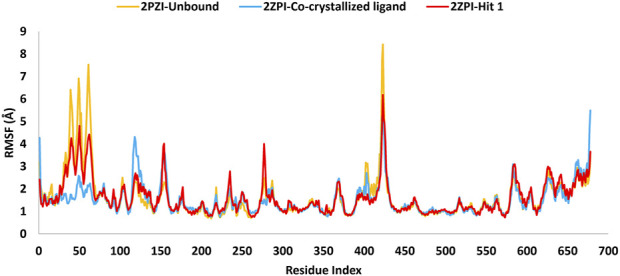
MD simulation trajectory analysis of RMSF of the C-α atoms of the unbound PknG (PDB: 2PZI) and its complexes with the co-crystallized ligand and Hit 1.

**FIGURE 15:**
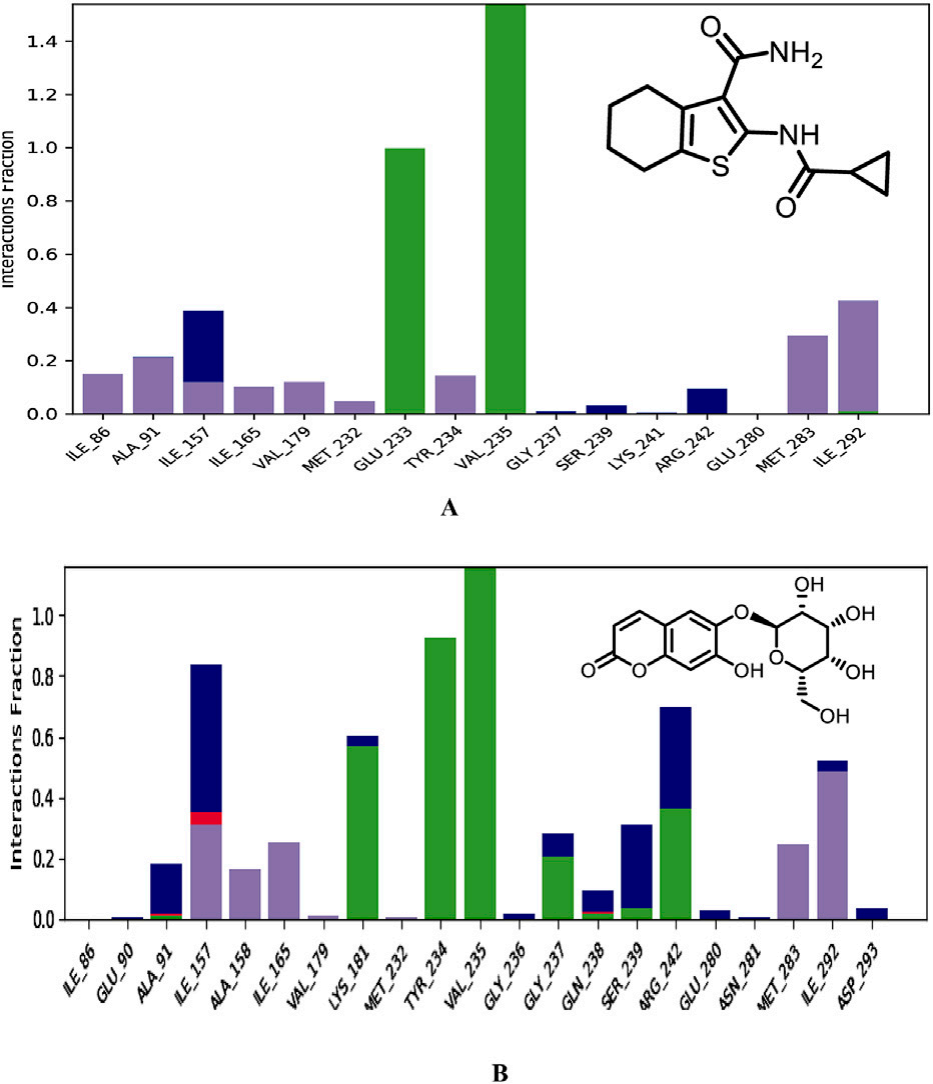
Binding modes of co-crystallized ligand **(A)**, Hit 1 **(B)** with PknG (PDB: 2PZI) during the simulation run. H-bonds are shown in green, hydrophobic interaction in grey, ionic bonds in deep pink and water bridges in blue.

To assess the strength of binding of the most promising compound, Hit **1,** to PknG binding site, all-atom MD simulation was performed for 100 ns time frame. The docked complexes of Hit 1 and the co-crystallized ligand were used as input files, alongside the unbound PknG for comparison. MD descriptors such as the Root Mean Squire Deviation (C-α RMSD) (Figure 13), Root Mean Squire of Fluctuation (RMSF) (Figure 14) and protein-ligand contacts (Figure 15) were extracted from the 100 ns trajectory simulation run. C-α RMSD calculations used extensively to evaluate the deviation of a protein relative to a reference structure during MD simulation. Low RMSD values ligand-protein complex indicate stability and limited structural changes (Arnittali et al., 2019). The RMSD plot (Figure 13) indicates that Hit **1** complex displayed relatively high fluctuation in the initial frames when compared to the co-crystallized ligand and the unbound protein. This could be attributed to the initial structural adjustments of Hit **1** complex to adopt the real binding mode. Following 10 ns of the MD simulation start, Hit **1** complex stabilization was attained and steady profile was displayed throughout the rest of simulation run. Generally, Hit **1** complex displayed great stability with minimal fluctuations (averaging RMSD 2.73 ± 0.37 Å) (Table 5) when compared to those of the co-crystallized ligand (averaging RMSD 3.03 ± 0.45 Å) and the unbound protein (averaging RMSD 3.52 ± 0.65 Å). This indicates that Hit **1** formed stable complex with PknG binding pocket and it remained tightly bound there for the whole of the simulation course.

**TABLE 5:** Detailed analysis of the RMSD values, including minimum, maximum, and average for the Hit 1 complex. This also includes comparative data for the co-crystallized ligand complex and the unbound protein.

PknG RMSD (Å)			
Protein	Maximum	Minimum	Average
2PZI Unbound	5.46	1.41	3.52 ± 0.65
2PZI-Co-crystallized ligand	4.63	1.57	3.03 ± 0.45
2PZI-Hit **1**	4.36	1.42	2.73 ± 0.37

The RMSF analysis presented in Table 6 provided insights into the dynamic flexibility of the PknG protein under different conditions. In its unbound state (2PZI), the protein exhibited the highest flexibility, with a maximum RMSF of 8.4 Å, a minimum of 0.68 Å, and an average of 1.61 Å, indicating significant motion within the structure. Binding with the co-crystallized ligand notably reduced the flexibility, reflected by a lower maximum RMSF of 5.4 Å, a minimum of 0.74 Å, and an average of 1.50 Å, suggesting enhanced stabilization of the protein. When bound to Hit **1**, the flexibility of PknG was intermediate, with a maximum RMSF of 6.1 Å, a minimum of 0.72 Å, and an average of 1.60 Å. This implied that Hit **1** provided some level of stabilization to the protein but was less effective compared to the co-crystallized ligand. Overall, the data indicated that while Hit **1** binding reduced the protein’s flexibility relative to its unbound state, it did not achieve the same level of stabilization as the co-crystallized ligand, highlighting its potential as a stabilizing agent but with scope for further optimization.

**TABLE 6:** Detailed analysis of the RMSF values, including minimum, maximum, and average for the Hit 1 complex. This also includes comparative data for the co-crystallized ligand complex and the unbound protein.

PknG RMSF (Å)			
Protein	Maximum	Minimum	Average
2PZI Unbound	8.4	0.68	1.61
2PZI-Co-crystallized ligand	5.4	0.74	1.50
2PZI-Hit 1	6.1	0.72	1.60

Moreover, an analysis of the flexibility of the binding site residues was performed. The RMSF analysis of the PknG binding site residues, revealed that flexibility was similar across the different conditions. In the unbound state (2PZI), the binding site exhibited a maximum RMSF of 1.6 Å, a minimum of 0.68 Å, and an average of 1.04 Å, indicating moderate motion. Binding with the co-crystallized ligand slightly increased the maximum RMSF to 1.67 Å and the minimum to 0.75 Å, while maintaining an average of 1.04 Å, suggesting minimal impact on the stabilization of the binding site residues. Binding with Hit **1** resulted in a maximum RMSF of 1.64 Å, a minimum of 0.80 Å, and a slightly higher average of 1.10 Å. These results suggested that both the co-crystallized ligand and Hit **1** influenced the dynamics of the binding site residues to a similar extent, with Hit **1** inducing slightly higher average flexibility. This indicated that Hit **1**’s binding effect was comparable to that of the co-crystallized ligand but required further optimization to achieve greater stabilization.

Near the conserved ATP-binding site, PknG possessed an additional hydrophobic pocket, distinct from most protein kinases due to its low sequence homology (Scherr et al., 2007; Caballero et al., 2018). This distinctiveness was attributed to the presence of a unique N-terminal segment spanning residues 5–60, which contributed significantly to the structural and functional divergence of PknG. The dynamic nature of this N-terminal segment was evident in the RMSF analysis, which revealed marked differences between the bound and unbound states. In the unbound state (2PZI), residues 5–60 exhibited the highest flexibility, with an RMSF range of 1.26–6.9 Å and an average of 3.03 Å, reflecting considerable motion within this region. Binding with the co-crystallized ligand markedly reduced flexibility, with an RMSF range of 1.28–2.5 Å and an average of 1.68 Å, indicating strong stabilization of these residues. In comparison, binding with Hit 1 resulted in intermediate flexibility, with an RMSF range of 1.21–4.8 Å and an average of 2.4 Å. While Hit **1** reduced the flexibility of residues 5–60 compared to the unbound state, it did not provide the same degree of stabilization as the co-crystallized ligand. These findings highlighted the potential of Hit **1** as a stabilizing agent for this region, though further optimization may be required to enhance its effectiveness. The significant reduction in RMSF values upon binding to the co-crystallized ligand or Hit **1** demonstrated that the hydrophobic pocket stabilized through ligand-induced structural adjustments. This stabilization not only enhanced binding affinity but also underscored the critical role of the N-terminal segment in regulating access to the hydrophobic pocket and optimizing ligand interactions. Notably, 90% of the inhibitors interacted with the unique N-terminal segment of PknG via hydrophobic interactions, further reinforcing the functional importance of this region (Caballero et al., 2018).

Plots for RMSF of amino acid residues are shown at a time function of 100 ns in Figure 14. It is clear that the key residues involved in protein-ligand interactions were of minimal flexibility (<1.5 Å) during the entire trajectory run. These low RMSF values for the interacting residues significantly impacted the stability and the firm binding of Hit **1** to the enzyme catalytic site. Further, relatively high variations were observed for residues Pro99 to Ser133, Arg222, Asn346 and Ala490 to Gly495 for both, the unbound protein and Hit **1** complex. Fortunately, these residues did not play a direct role in interacting with Hit **1**, so their flexibility did not impact the overall stability of Hit **1** complex.

Next, different types of protein-ligand interactions occurring in the simulation time were investigated. Four different types of protein-ligand contacts were identified, specifically: H-bonds, water bridges, ionic interactions, and hydrophobic interactions. As illustrated in Figure 15, the co-crystallized ligand maintained the same interactions with the key residues encountered for XP docking conformer. These interactions included three H-bonds with the hinge region residues, two with Val235 and the other with Glu233. These H-Bonds retained for the entire simulation time frame. Further, hydrophobic interactions were also observed with critical residues Ile86, Ala91, Ile157, Ile165, Val179, Met232, Tyr234, Met283, and Ile292. The hinge region of PknG, comprising Glu233, Tyr234, and Val235, plays a pivotal role in ligand stabilization (Arica-Sosa et al., 2022). In this context, the hydrogen bond formed by Hit **1** with the key residue Val235 in this region remained stable for the majority of the simulation time (93%), compared to 99% stability observed in the co-crystallized ligand complex (Supplementary Figure S3). However, the H-Bond with the crucial residue Glu233 detected for the docked complex lost and was replaced with stable H-Bond with the adjacent residue Tyr234 in the same region. The later bond was also maintained for most of the simulation time (92%). This loss of interaction with Glu233 may impact the overall binding affinity, as Glu233 plays a significant role in stabilizing ligands within the ATP-binding site. However, Hit **1** compensated for this loss by forming a stable interaction with the hinge region residue Tyr234 and the catalytic residue Lys181 (Arica-Sosa et al., 2022), which were present for nearly 92% and 60% of the simulation, respectively. These interactions, which were not observed for the co-crystallized ligand, may contribute to maintaining binding stability and potentially offset the impact of the missing Glu233 interaction. Given that Lys181 is critical for catalytic activity, the interaction with this residue could enhance the functional efficacy of Hit **1**, despite the reduced affinity caused by the absence of Glu233 engagement. Thus, Future optimization of Hit **1** could focus on re-establishing interactions with Glu233 to improve its binding affinity without compromising its interaction with Lys181. In addition, Hit **1** created multiple H-Bonds and Water Bridges with different residues including Lys181, Ile157, Ser239, and Arg242. Some of these bonds lasted for >55% of the timeline, further contributed to stabilization of Hit **1** complex. These additional connections could be attributed to the presence of the sugar moiety attached to C-6 of the chromone ring in Hit **1** which amplifies its hydrophilicity as compared to the co-crystallized ligand. Regarding hydrophobic interaction, it was observed for Hit **1** with several residues existed in the hydrophobic region of the enzyme binding pocket such as Ile157, Ala158, Ile165, Val179, Met283, and Ile292.

Furthermore, to get detailed insights into the stability and specificity of the PknG-Hit **1** interactions, the number of H-Bonds along with hydrophobic interactions were analyzed and the results are provided in Table 7 and Supplementary Figure S4. Hit **1** demonstrated a relatively high number of H-Bonds during the simulation compared to those made by the co-crystallized ligand (Supplementary Figure S4A), averaging 3.28 and 2.54, respectively. This could justify the better stability of Hit **1** complex compared to that of the co-crystalized ligand, as H-bonds are increasingly regarded as facilitators of protein-ligand binding (Chen et al., 2016). Moreover, Hit **1** established hydrophobic interactions (Supplementary Figure S4B), comparable to those formed by the co-crystallized ligand, with averages 1.47 and 1.61, respectively. Thus, we can reasonably conclude that Hit **1** created a stable and favorable interaction profile with the mycobacterial PknG catalytic site, making it a potential PknG inhibitor for the treatment of tuberculosis. The average water-bridge contacts for the co-crystallized ligand were 0.41, while Hit **1** showed a significantly higher average of 1.55. This suggested that Hit **1** formed more frequent water-mediated interactions with the protein. The maximum number of water-bridge contacts for Hit **1** was 7, compared to 4 for the co-crystallized ligand, indicating that Hit **1** had the potential to establish stronger networks. Both ligands showed a minimum of 0 water bridges, implying the absence of such interactions in some configurations. These findings highlighted the enhanced water-bridge interaction of Hit **1** with the protein. In conclusion, Hit **1** demonstrated a more favorable interaction profile with PknG, evidenced by a higher number of hydrogen bonds and water-bridge contacts, as well as comparable hydrophobic interactions to the co-crystallized ligand. These factors suggest that Hit **1** formed a more stable and specific complex with the mycobacterial PknG catalytic site. Therefore, Hit **1** presents itself as a promising candidate for further optimization as a PknG inhibitor for tuberculosis treatment.

**TABLE 7:** Maximum, Minimum and Average number of H-Bonds, hydrophobic interactions, and Water-Bridges observed during 100 ns MD simulation of hit 1 and the co-crystallized ligand complexes with PknG (PDB: 2PZ1).

2PZI-complex	Co-crystallized ligand	Hit 1
H-bond contacts		
Average	2.54	3.28
Maximum	4	8
Minimum	1	0
Hydrophobic contacts		
Average	1.61	1.41
Maximum	7	5
Minimum	0	0
Water-bridge contacts		
Average	0.41	1.55
Maximum	4	7
Minimum	0	0

### 3.6 Study limitations and future perspective

Various computational approaches, including molecular docking, free energy calculations, ADMETox studies, DFT analysis, and MD simulations, have been employed in this study to identify novel potential inhibitors targeting PknG of *M. tuberculosis*. These inhibitors are expected to suppress the growth and multiplication of drug-resistant *M. tuberculosis* that survive within alveolar macrophages, thereby potentially addressing the challenge of latent tuberculosis. A compound library comprising 460,000 small molecules underwent multimode virtual screening, from which seven compounds (hits 1–7) demonstrated notable binding affinity and robust interactions with essential residues within the catalytic binding pocket of mycobacterial PknG. While all seven hits exhibited acceptable drug-like properties, the chromene glycoside (hit 1) stood out for its lower toxicity. However, strategies to optimize its oral bioavailability are strongly recommended. Additionally, DFT analysis indicated that the mechanical and electronic properties of hit 1 were superior to those of the reference ligand (AX20017). Molecular dynamics (MD) simulations further confirmed the stability of the hit 1-PknG complex, supporting its potential as a lead compound for anti-tuberculosis drug development. Despite these promising computational results, several limitations must be acknowledged. The reliability of computational models depends heavily on the quality of structural data and the assumptions used in the simulations, which, although thorough, may not fully capture the intricate complexities of biological systems *in vivo*. In silico models, while powerful, have inherent limitations in accurately predicting the behavior of compounds within living organisms. These models often fail to account for complex *in vivo* molecular interactions, such as off-target effects and biotransformation pathways, introducing uncertainty regarding the safety and efficacy of the investigated compounds. Molecular docking, a fundamental computational technique, relies on assumptions about the flexibility of proteins and ligands. It typically represents the binding site as static or semi-flexible, potentially overlooking the dynamic nature of proteins that can adopt multiple conformations. Consequently, docking scores, which estimate binding potential, do not always correlate with experimental binding affinity values. To mitigate these issues, MM-GBSA calculations were employed to provide more accurate binding energy estimates by incorporating molecular flexibility and solvent effects. Additionally, MD simulations validated the stability of PknG-inhibitor complexes, offering dynamic insights and reinforcing the potential of the identified hit as effective PknG inhibitors. Nonetheless, this study’s structure-based virtual screening was limited to the NCI database, which, despite its extensive coverage, may exclude certain chemical classes with potential PknG inhibitory activity. Future studies should explore additional chemical libraries to identify a broader range of inhibitors. Furthermore, translating computational findings into actionable drug discovery requires experimental validation. Wet lab investigations, such as enzyme inhibition assays and cell-based activity tests, are essential to confirm the identified hit’s activity. Additionally, *in vivo* studies are necessary to evaluate the ADMET profile, therapeutic potential, and overall safety of the identified hit. Structural modifications to the lead candidate, chromene glycoside, or the implementation of various formulation and delivery strategies should also be explored to address its bioavailability limitations while preserving its inhibitory potency.These efforts are crucial to bridging the gap between computational predictions and clinical applications, ensuring the development of effective PknG inhibitors for tuberculosis treatment.

## 4 Conclusion

In conclusion, this study employed a comprehensive array of computational approaches to identify potential inhibitors targeting PknG of M.tb. Among the seven promising hits identified, chromene glycoside (hit 1) emerged as the most potent candidate due to its strong binding affinity, favorable interactions, and lower toxicity profile. DFT analysis further highlighted its superior electronic properties compared to the reference ligand. Molecular dynamics simulations confirmed the stability of the hit 1-PknG complex, reinforcing its potential as a lead compound. However, optimization of its oral bioavailability remains necessary to enhance its drug-like properties and therapeutic applicability.

## Data Availability

The original contributions presented in the study are included in the article/Supplementary Material, further inquiries can be directed to the corresponding authors.
