# Supplementary material for: Molecular docking, free energy calculations, ADMETox studies, DFT analysis, and dynamic simulations highlighting a chromene glycoside as a potential inhibitor of PknG in Mycobacterium tuberculosis
Source: Front Chem. 2025 Feb 25;13:1531152. doi: 10.3389/fchem.2025.1531152 (PMC11893855; doi:10.3389/fchem.2025.1531152)
Supplement: Supplementary file 1 [file DataSheet1.docx]

**Molecular Docking, Free Energy Calculations, ADMETox Studies, DFT Analysis, and Dynamic Simulations Highlighting a Chromene Glycoside as a Potential Inhibitor of PknG in *Mycobacterium tuberculosis***

**Muharib Alruwaili*^1^, Tilal Elsaman*^2^, Magdi Awadalla Mohamed^2^, Abozer Y. Elderdery^1^, Jeremy Mills^3^, Yasir Alruwaili^1^, Siddiqa M. A. Hamza^4^, Salma Elhadi Ibrahim Mekki^5^, Hazim Abdullah Alotaibi^6^, Maily J. Alrowily^7^ and Maryam Musleh Althobiti^8^**

^1^Department of Clinical Laboratory Sciences, College of Applied Medical Sciences, Jouf University, Sakaka, SaudiArabia,

^2^Department of Pharmaceutical Chemistry, College of Pharmacy, Jouf University, Sakaka, Saudi Arabia.

^3^School of Pharmacy and Biomedical Sciences, University of Portsmouth, Portsmouth, United Kingdom,

^4^Department of Pathology, College of Medicine in Alqunfudah, Umm Alqura University, Algunfuda, SaudiArabia,

^5^Department of Physiology, College of Medicine in Alqunfudah, Umm Alqura University, Alqunfudah, Saudi Arabia,

^6^Departmentof Internal Medicine and Oncology, Prince Mohammed Medical City, Hail, SaudiArabia,

^7^Consultant-Research Center, Aljouf Health Cluster, Aljouf, SaudiArabia,

^8^Department of Clinical Laboratory Sciences, College of Applied Medical Sciences, Shaqra University, Shaqra, Saudi Arabia

Received: / Accepted: / Published:

*Authors to whom correspondence should be addressed; [telbashir@ju.edu.sa](mailto:telbashir@ju.edu.sa) and [mfalrwaili@ju.edu.sa](mailto:mfalrwaili@ju.edu.sa)

**Table S1: ADMET and drug-like properties of the top 7 hits determined by Qikprop module of Schrödinger**

| **Descriptor** | **Description** | **Permissible range** | **INH** | **1** | **2** | **3** | **4** | **5** | **6** | **7** |
| --- | --- | --- | --- | --- | --- | --- | --- | --- | --- | --- |
| #stars | Descriptor values number that fall outside the 95% range of similar values for known drugs. | (0–5) | 1 | 0 | 0 | 0 | 0 | 0 | 1 | 0 |
| #amide | Non-conjugated amide groups number | (0–1) | 0 | 0 | 0 | 0 | 0 | 0 | 0 | 0 |
| #rotor | Number of bonds for both non-trivial, non-hindered rotatable | (0–15) | 2 | 8 | 4 | 3 | 1 | 6 | 3 | 4 |
| #rtvFG | Reactive functional groups number | (0–2) | 1 | 2 | 0 | 0 | 1 | 0 | 1 | 0 |
| mol_MW | Molecular weight (MW) of the molecule. | (130–725) | 137.141 | 340.286 | 385.397 | 351.425 | 366.394 | 363.405 | 342.356 | 364.421 |
| SASA | Total solvent accessible surface area | (300–1000) | 329.652 | 540.781 | 666.32 | 643.469 | 590.844 | 602.749 | 570.239 | 658.748 |
| FOSA | Hydrophobic component of the SASA | (0–750) | 0 | 118.685 | 184.448 | 178.173 | 173.479 | 0 | 72.388 | 121.756 |
| FISA | Hydrophilic component of the SASA | (7–330) | 164.330 | 277.916 | 161.885 | 82.508 | 55.888 | 260.57 | 46.718 | 109.148 |
| PISA | π (carbon and attached hydrogen) component of the SASA | (0–450) | 164.330 | 144.18 | 283.16 | 345.985 | 316.058 | 287.74 | 451.133 | 389.662 |
| WPSA | Weakly polar component of the SASA | (0–175) | 0 | 0 | 36.827 | 36.804 | 45.42 | 54.439 | 0 | 38.183 |
| donorHB | Estimated number of hydrogen bonds that would be donated by the solute to the water molecules of an aqueous solution. | (0–6) | 3 | 5 | 1 | 1 | 1 | 5 | 1 | 2 |
| accptHB | Estimated number of hydrogen bonds that would be accepted by the solute from water molecules in an aqueous solution | (2–20) | 4.5 | 12.5 | 6.5 | 6.5 | 6.5 | 8 | 5.5 | 5.75 |
| QPlogPo/w | Predicted octanol/water partition coefficient. | (−2–6.5) | ‒0.647 | ‒1.531 | 3.049 | 3.485 | 3.25 | 0.641 | 3.552 | 3.648 |
| QPPCaco | Predicted apparent Caco-2 cell permeability in nm/sec | (<25poor, >500 great) | 273.879 | 22.931 | 288.9 | 1634.854 | 2923.583 | 33.49 | 3571.664 | 913.797 |
| QPlogS | Predicted aqueous solubility | –6.5 – 0.5 | ‒1.034 | ‒1.776 | -5.523 | -5.288 | ‒4.744 | ‒3.67 | ‒4.218 | ‒5.636 |
| CIQPlogS | Conformation-independent predicted aqueous solubility | –6.5 – 0.5 | ‒1.034 | ‒2.122 | ‒5.405 | ‒4.783 | ‒5.197 | ‒4.276 | ‒5.414 | ‒5.294 |
| QPlogBB | Predicted brain/blood partition coefficient. | –3.0 – 1.2 | ‒0.847 | ‒2.386 | ‒1.308 | ‒0.376 | 0.086 | ‒2.277 | ‒0.044 | ‒0.734 |
| QPPMDCK | Predicted apparent MDCK cell permeability in nm/sec. | <25 poor >500 great | 122.016 | 8.359 | 205.693 | 1338.769 | 2797.428 | 25.014 | 1958.564 | 726.437 |
| #metab | Number of likely metabolic reactions. | (1 – 8) | 2 | 5 | 3 | 4 | 2 | 3 | 2 | 5 |
| QPlogKhsa | Prediction of binding to human serum albumin. | −1.5–1.5 | ‒0.754 | ‒0.978 | 0.256 | 0.224 | 0.144 | ‒0.439 | 0.191 | 0.35 |
| QPlogHERG | Predicted IC_50_ value for blockage of HERG K^+^ channels. | Concern below –5 | ‒3.591 | ‒4.379 | ‒6.25 | ‒6.336 | ‒5.638 | ‒5.891 | ‒6.032 | ‒6.699 |
| Human Oral Absorption | Predicted qualitative human oral absorption | 1, 2, or 3 for low, medium, or high. | 2 | 2 | 3 | 3 | 3 | 2 | 3 | 3 |
| %Human Oral Absorption (PHOP%) | Predicted human oral absorption on 0 to 100% scale. | >80% is high | 66.7 | 42.331 | 88.843 | 100 | 100 | 57.993 | 100 | 100 |
| PSA | VdW surface area of polar nitrogen and oxygen atoms | 7–200 | 81.5 | 164.524 | 109.356 | 67.533 | 73.333 | 128.997 | 58.318 | 77.035 |
| Rule Of Five | Number of violations of Lipinski’s rule of five | maximum is 4 | 0 | 0 | 0 | 0 | 0 | 0 | 0 | 0 |
| Rule Of Three | Number of violations of Jorgensen’s rule of three. | maximum is 3 | 0 | 1 | 0 | 0 | 0 | 0 | 0 | 0 |

**Table S2: The predicted output values of the toxicological profiles of the top 7 hits as determined by ADMETLab 2.**

| **Property** | **Description** | **Empirical decision** | **INH** | **1** | **2** | **3** | **4** | **5** | **6** | **7** |
| --- | --- | --- | --- | --- | --- | --- | --- | --- | --- | --- |
| H-HT | The human hepatotoxicity | 0-0.3: excellent; 0.3-0.7: medium; 0.7-1.0: poor | 0.71 | 0.129 | 0.846 | 0.831 | 0.743 | 0.128 | 0.786 | 0.577 |
| DILI | Drug-induced liver injury | 0-0.3: excellent; 0.3-0.7: medium; 0.7-1.0: poor | 0.70 | 0.818 | 0.98 | 0.984 | 0.974 | 0.99 | 0.979 | 0.902 |
| AMES Toxicity | test for mutagenicity | 0-0.3: excellent; 0.3-0.7: medium; 0.7-1.0: poor | 0.93 | 0.339 | 0.997 | 0.725 | 0.936 | 0.177 | 0.472 | 0.932 |
| ROA | Determination of oral acute toxicity in rats | 0-0.3: excellent; 0.3-0.7: medium; 0.7-1.0: poor | 0.58 | 0.041 | 0.023 | 0.051 | 0.018 | 0.411 | 0.462 | 0.019 |
| FDAMDD | The maximum recommended daily dose provides an estimate of the toxic dose threshold of chemicals in humans | 0-0.3: excellent; 0.3-0.7: medium; 0.7-1.0: poor | 0.062 | 0.009 | 0.086 | 0.034 | 0.385 | 0.877 | 0.586 | 0.677 |
| SkinSen | Skin Sensitization | 0-0.3: excellent; 0.3-0.7: medium; 0.7-1.0: poor | 0.98 | 0.695 | 0.242 | 0.321 | 0.133 | 0.044 | 0.685 | 0.233 |
| Carcinogenicity | The ability to damage the genome or disrupt cellular metabolic processes | 0-0.3: excellent; 0.3-0.7: medium; 0.7-1.0: poor | 0.94 | 0.426 | 0.864 | 0.091 | 0.869 | 0.569 | 0.652 | 0.661 |
| EC | Assessing the eye corrosion (EC) potential of a chemical as a necessary component of risk assessment | 0-0.3: excellent; 0.3-0.7: medium; 0.7-1.0: poor | 0.30 | 0.011 | 0.003 | 0.003 | 0.003 | 0.003 | 0.003 | 0.003 |
| EI | Assessing the eye Irritation (EI) potential of a chemical as a necessary component of risk assessment | 0-0.3: excellent; 0.3-0.7: medium; 0.7-1.0: poor | 1 | 0.234 | 0.04 | 0.037 | 0.024 | 0.521 | 0.235 | 0.104 |
| Respiratory Toxicity | Assessment of the potential of Drug-induced respiratory toxicity | 0-0.3: excellent; 0.3-0.7: medium; 0.7-1.0: poor | 0.99 | 0.033 | 0.912 | 0.954 | 0.908 | 0.504 | 0.934 | 0.916 |

| **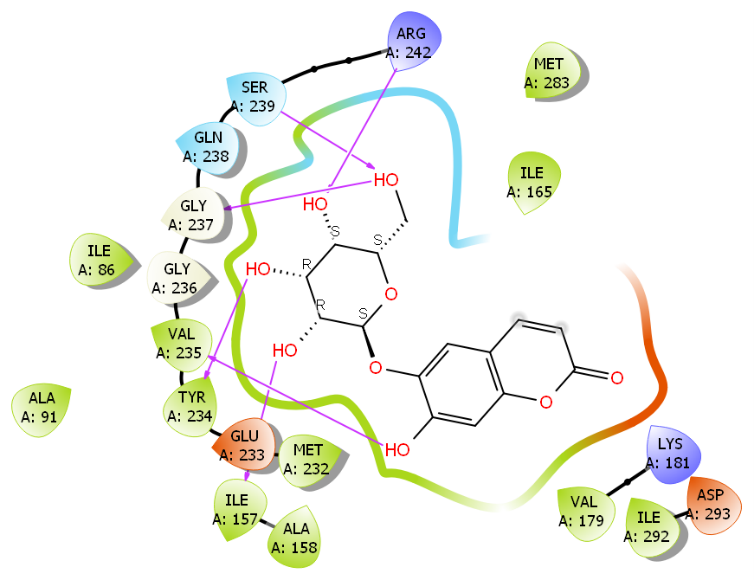** | **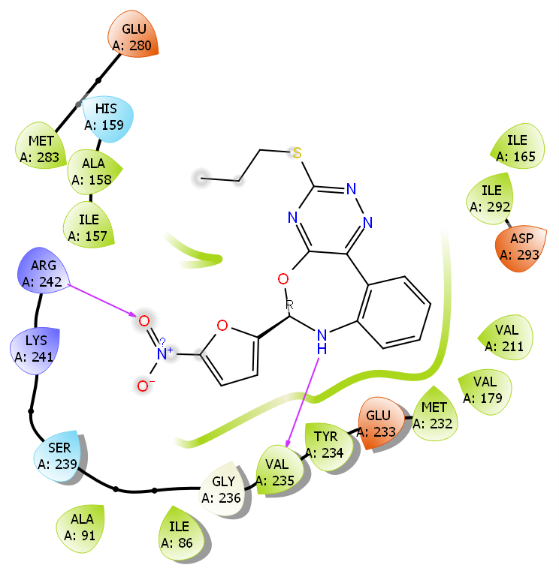** | **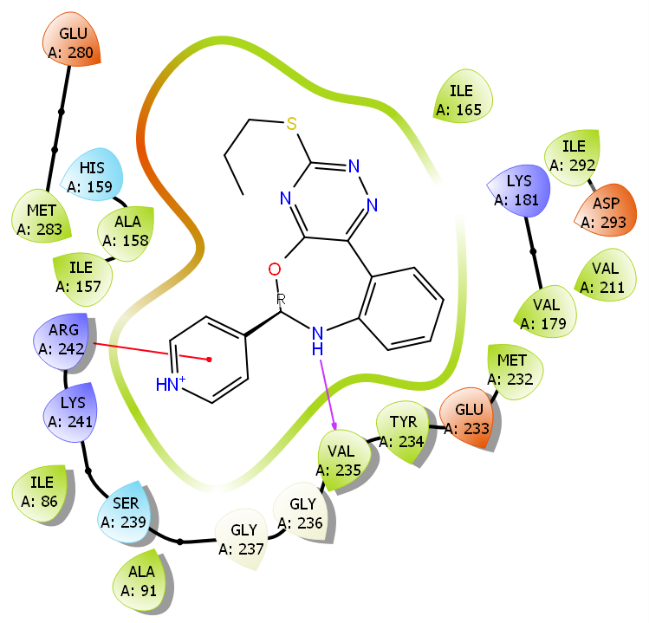** |
| --- | --- | --- |
| **Hit 1** | **Hit 2** | **Hit 3** |
| **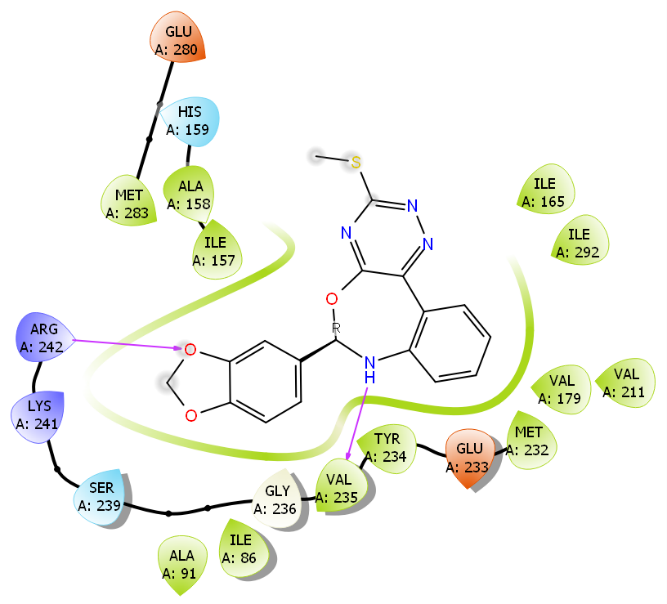** | **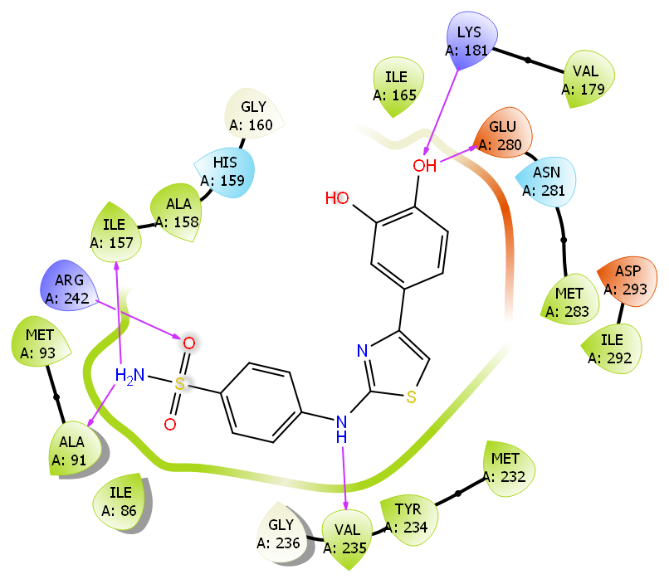** | **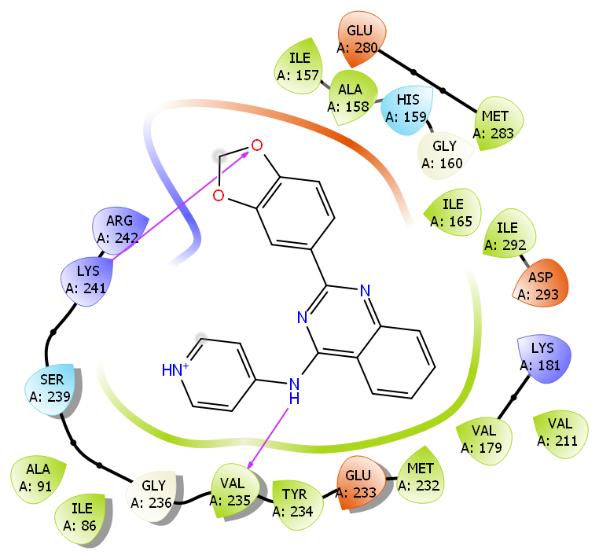** |
| **Hit 4** | **Hit 5** | **Hit 6** |
| **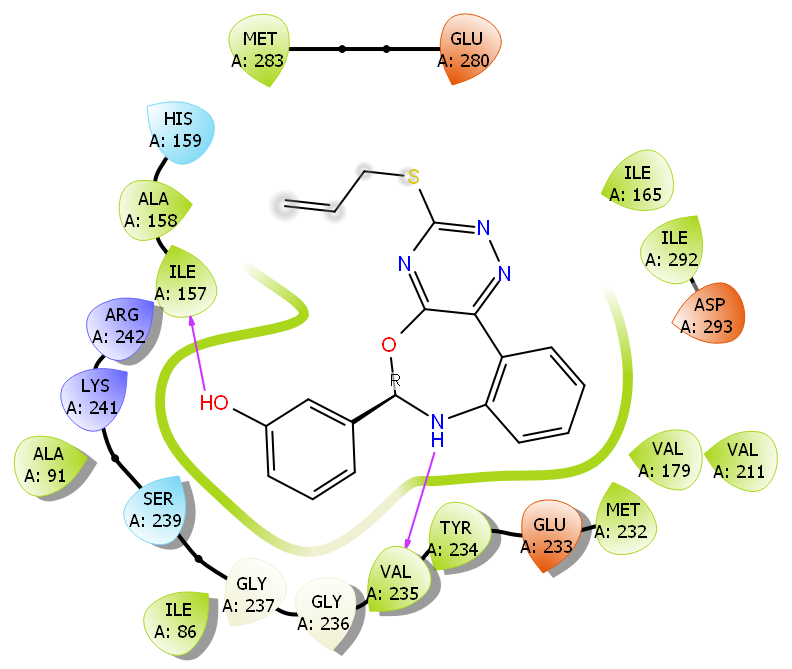** | | |
| **Hit 7** | | |
| 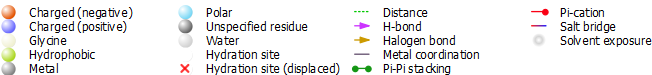 | | |

**Supplementary Figure S1. 2D interactions of hits 1-7**

| **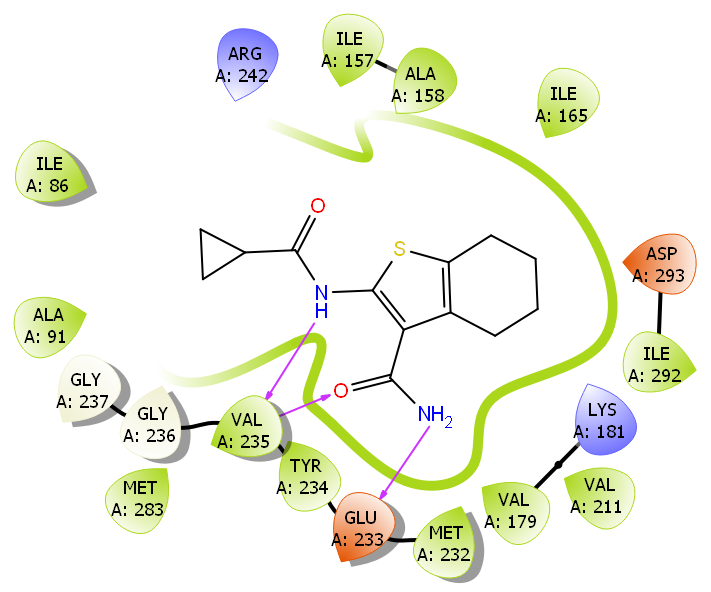** | 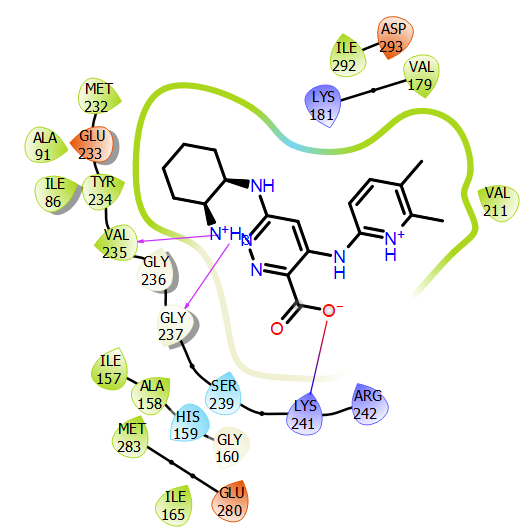 | 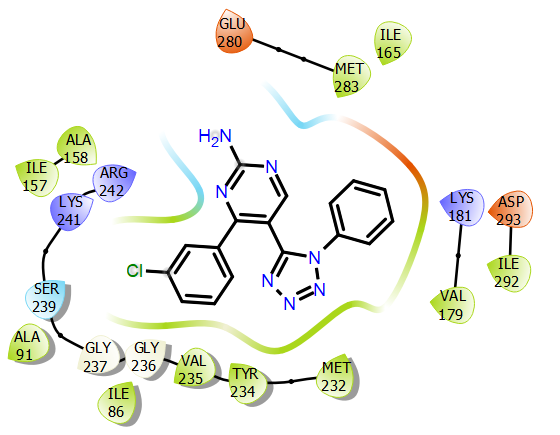 |
| --- | --- | --- |
| **AX20017** | **RO9021** | **NRB04248** |
| 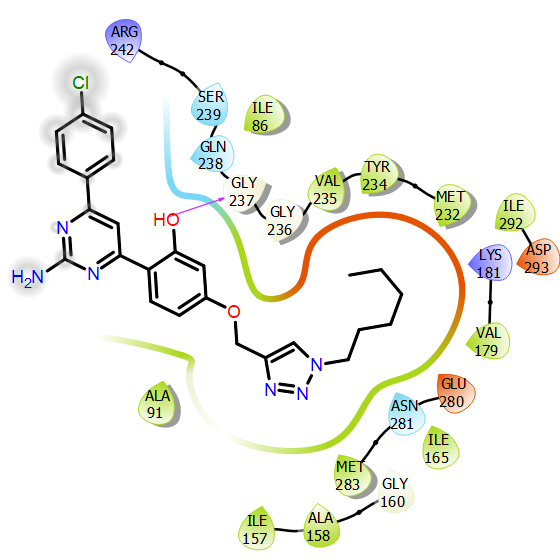 | 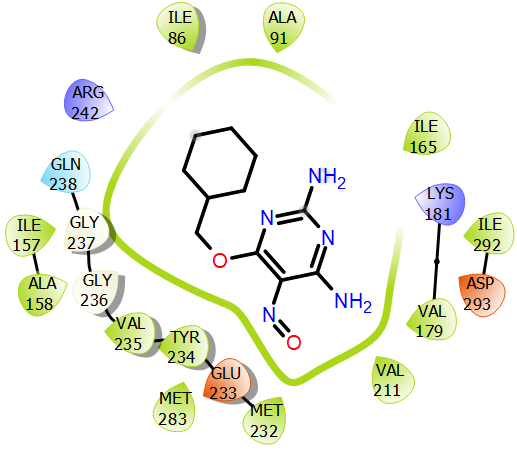 | 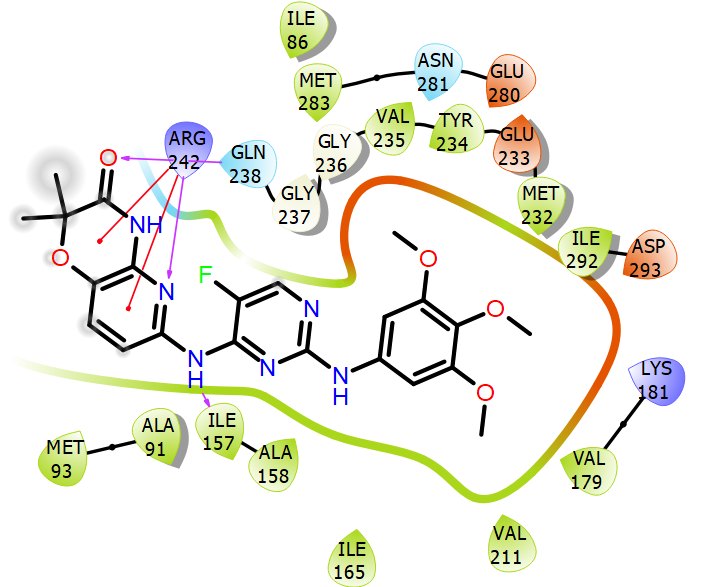 |
| **Aminopyrimidine derivative** | **NU-6027** | **R406** |
| 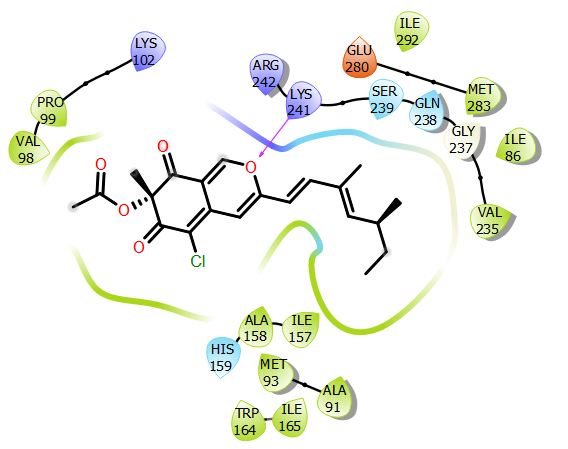 |  |  |
| **Sclerotiorin** |  |  |
| 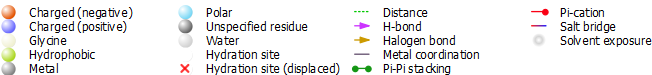 | | |

**Supplementary Figure S2. 2D interactions of the experimentally validated M.tb PknG inhibitors with the target enzyme (PDB ID: 2PZI)**


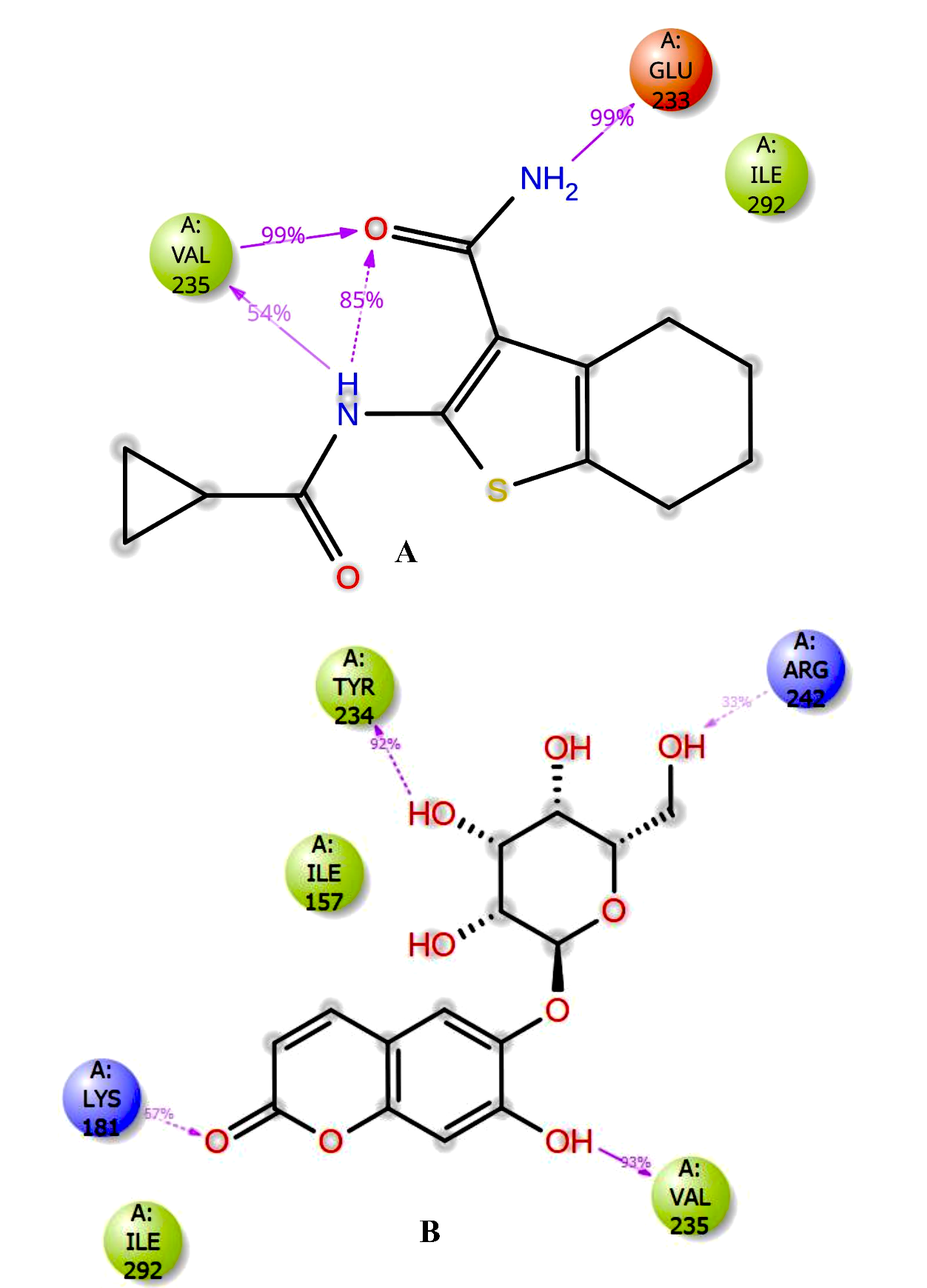


**Supplementary Figure S3. Interaction timeline of co-crystallized ligand and hit 1 with Pkng (2PZI) during 100 ns molecular dynamics simulation.**


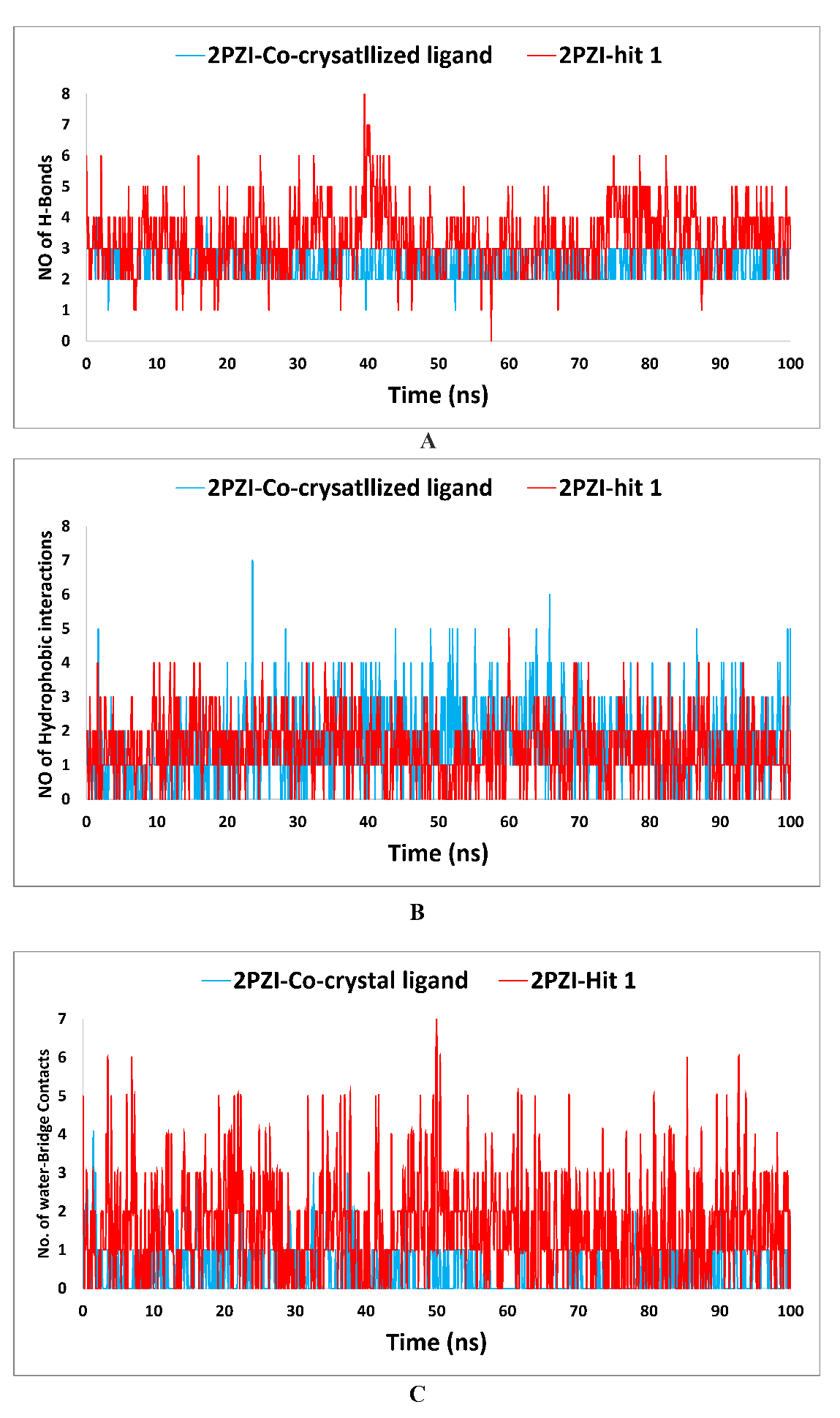


Supplementary Figure S4: Number of H-Bonds (A), Hydrophobic interactions (B), and Water-Bridges (C) formed during 100 ns MD simulation of hit 1 and the co-crystallized ligand complexes with PknG (PDB: 2PZ1)
